# Sex and ovarian hormone cycles alter effects of stimulant drugs on mouse dopaminergic signaling

**DOI:** 10.1172/JCI178630

**Published:** 2026-03-17

**Authors:** Brooke A. Christensen, Jennifer Tat, Michael Z. Leonard, Soren D. Emerson, Shemuel Roberts, Eleanor B. Holmgren, Ainoa Konomi-Pilkati, Hannah B. Reiley, Devan M. Gomez, Lin Zheng, Hye Jean Yoon, Sofia H. Lago, Abigail L. Carr, Lillian J. Brady, Maxime Chevée, Erin S. Calipari

**Affiliations:** 1 Department of Pharmacology,; 2 Vanderbilt Center for Addiction Research,; 3 Vanderbilt Brain Institute, Vanderbilt University, Nashville, Tennessee, USA.; 4 Department of Neuroscience, University of Copenhagen, Copenhagen, Denmark.; 5 Department of Psychiatry and Behavioral Neurobiology, Heersink School of Medicine, The University of Alabama at Birmingham, Birmingham, Alabama, USA.; 6 Department of Molecular Physiology and Biophysics and; 7 Department of Psychiatry and Behavioral Sciences, Vanderbilt University, Nashville, Tennessee, USA.

**Keywords:** Cell biology, Neuroscience, Addiction, Behavior

## Abstract

Stimulant medications are widely prescribed for attention deficit hyperactivity disorder (ADHD) and have significant abuse liability. Here, we show that, consistent with clinical data, female mice exhibited enhanced behavioral sensitivity to stimulants, and we define sex- and hormone-dependent adaptations in the dopamine system that contributed to these effects. Single-nucleus RNA-seq of ventral tegmental area dopamine neurons revealed that projections to the nucleus accumbens (NAc) core, compared with other projection populations, were a hub of sexually dimorphic gene expression, including transcripts regulating dopamine synthesis, and transport. These molecular differences coincided with enhanced dopamine release and clearance in female mice, particularly during phases of the estrous cycle when estradiol levels were high. The stimulants amphetamine (a releaser) and methylphenidate (a reuptake inhibitor) more effectively increased dopamine levels in female mice under certain conditions. However, amphetamine showed more robust hormone-sensitive regulation, with potency reduced by ovariectomy and restored by direct estradiol replacement in the NAc core. Together, the findings indicate that even within a drug class, drugs with different mechanisms of action can leverage different aspects of sexually dimorphic dopamine function. This distinction highlights the notion that sex differences are not uniform but can be differentially sensitive to drug pharmacology.

## Abstract 2

Biological sex and ovarian hormones shape responses to stimulant medications by altering dopamine signaling. Drug effects vary by sex, hormonal state, and mechanism of action.

## Introduction

Stimulant medications are some of the most widely prescribed medications in the United States and the rate of prescription has risen dramatically in recent years (1). Prescriptions for a range of medications to treat attention deficit hyperactivity disorder (ADHD) increased 45% between 2012 and 2021 (2). Amphetamine (AMPH) prescriptions (Adderall and its generic versions) increased by more than 58% for adults from 2018 to 2022 (3). Beyond clinical use, these drugs are frequently misused, with more than 300,000 adults initiating prescription-stimulant misuse in 2021 alone (4).

Despite extensive research on these medications, there has been limited investigation into how biological sex influences their pharmacodynamic actions—a critical knowledge gap given emerging evidence of sexually dimorphic stimulant responses (5–9). Women and men show distinct patterns of drug effects on behavior (5), treatment efficacy for ADHD (6), side effect profiles of stimulant medications (7, 8), and the expression of disorders for which these drugs are prescribed (9). Importantly, neuroimaging studies reveal that the stimulant methylphenidate produces greater dopamine transmission increases in the nucleus accumbens (NAc) of women compared with men (10), whereas AMPH effects appear more variable and potentially hormone cycle dependent (11, 12). These clinical observations suggest fundamental sex differences in how stimulants interact with the dopamine system, yet the underlying mechanisms remain poorly understood.

Understanding these sex differences requires examining how stimulants act on their primary target: the dopamine system (13). Dopamine is released into the synaptic space, and this signal is terminated via clearance through the dopamine transporter (DAT) (13, 14). Stimulants exert their therapeutic effects, in part, through elevating dopamine levels in the NAc (11, 12, 15) via actions at the DAT. Reuptake inhibitors, like methylphenidate, inhibit DAT, preventing dopamine clearance, which leads to its accumulation in the synaptic space (13, 16). Releasers, like AMPH, both block DAT and actively promote dopamine efflux by disrupting cellular pH gradients and reversing transporter function (17–19). Both methylphenidate and AMPH have efficacy for clinical disorders characterized by dopamine dysfunction (20–23). These mechanistic differences between stimulant classes become particularly relevant when considering that dopamine system function varies both at baseline between sexes and cyclically within female rodents due to ovarian hormones (24). Estradiol is a key mediator of hormone cycle effects where it is known to enhance dopamine neuron excitability, increase dopamine release, and modulate DAT function (24–34). During the proestrus/estrus phases of the rodent estrous cycle, when estradiol levels peak, dopamine neurons become hyperexcitable and stimulant effects are enhanced (28, 30–32). This hormonal sensitivity could create a dynamic substrate that differentially influences stimulant efficacy depending on drug mechanism and hormonal state.

Using an integrated approach spanning behavioral, molecular, protein, and functional analyses, we demonstrate that biological sex influences stimulant responses through multiple complementary mechanisms. We show that sex differences in attention-related behavioral responses to AMPH parallel enhanced dopamine system function in female mice. Single-nucleus RNA-Seq (snRNA-Seq) reveals that dopamine projections to the NAc core—rather than shell—represent a key hub of sexually dimorphic gene expression. Functional studies using fast-scan cyclic voltammetry (FSCV) demonstrate that enhanced dopamine release and clearance in female mice create a substrate for increased stimulant sensitivity, with ovarian hormones playing distinct roles in AMPH versus methylphenidate effects. Finally, ovariectomy and ex vivo hormone rescue experiments directly establish estradiol as a critical mediator of sex differences in AMPH potency, specifically. Together, this work provides mechanistic understanding of how biological sex and hormonal cycles influence stimulant-medication efficacy.

## Results

### AMPH enhanced performance in behavioral tasks associated with sustained attention to a greater extent in female mice.

**Figure 1:**
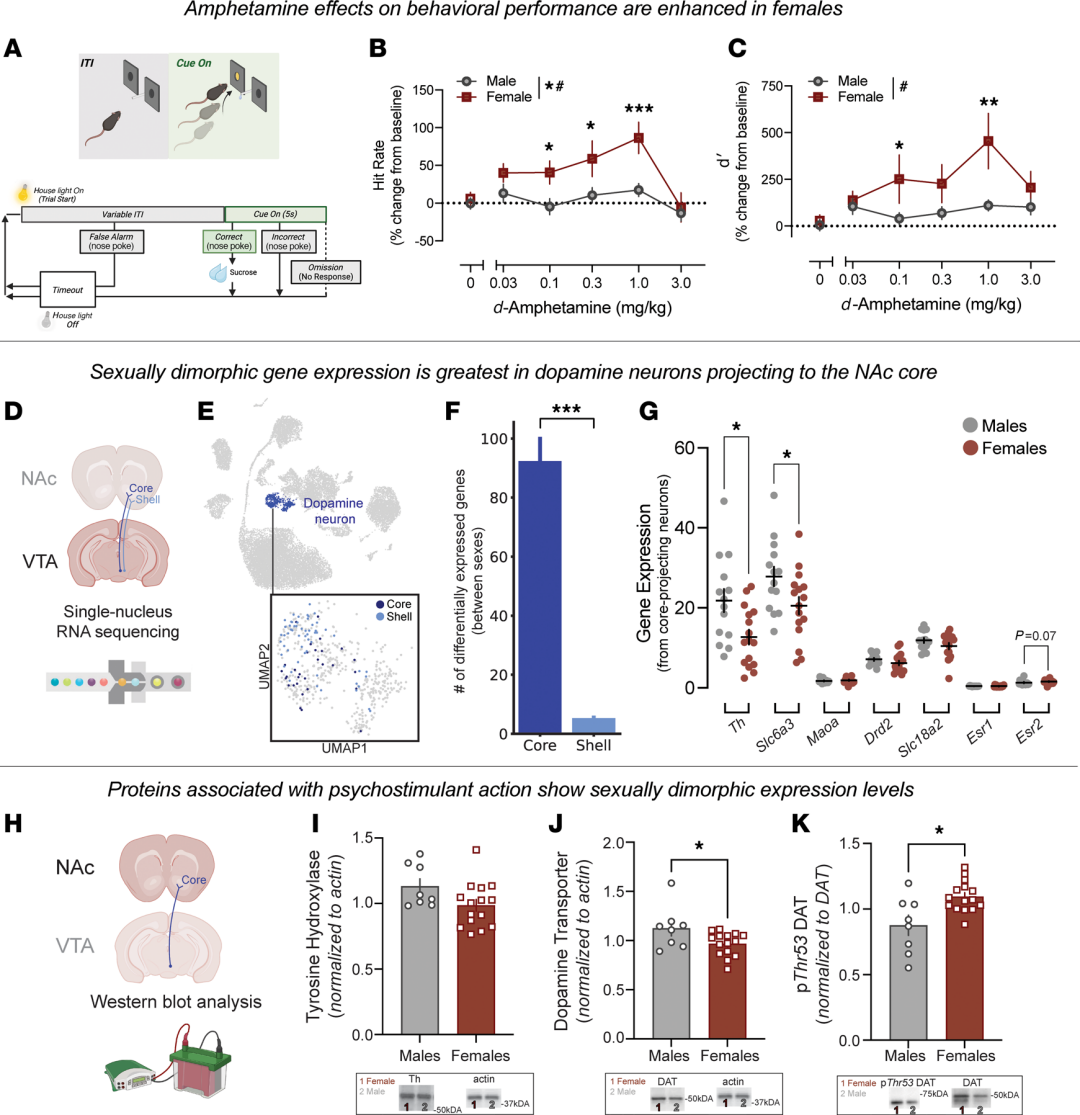
Sexual dimorphism in dopamine neurons projecting to the NAc accompanies enhanced AMPH sensitivity in female mice. (**A**) Mice performed a psychomotor vigilance task. (**B** and **C**) AMPH effects on sustained attention were measured as percent change from baseline in hit rate (**B**) and d′ (**C**). Female mice showed greater sensitivity to AMPH [hit rate: main effect of AMPH, *F*(5,105) = 9.48, *P* < 0.0001; main effect of sex, *F*(1,21) = 5.38, *P* = 0.031; interaction, *F*(5,105) = 2.97, *P* = 0.0150; d′: main effect of AMPH, *F*(5,105) = 5.32, *P* = 0.0002; sex × AMPH interaction, *F*(5,105) = 2.70, *P* = 0.024]. *Denotes significance between male and female mice. (**D**–**G**) snRNA-Seq of VTA dopamine neurons revealed more sex-DEGs in NAc core- versus shell-projecting neurons (Mann-Whitney *U* test: *U* = 2492.0, *P* < 0.0001). (**F**) Core-projecting neurons showed sex differences in key genes involved in dopamine synthesis (**G**): *Th* [unpaired *t* test: *t*(28) = 2.73, *P* = 0.011], *Slc6a3* [unpaired *t* test: *t*(28) = 2.25, *P* = 0.033], *Maoa* [unpaired *t* test: *t*(28) = 0.648; *P* = 0.522], *Drd2* [unpaired *t* test: *t*(28) = 1.282, *P* = 0.210], *Slc18a2* [unpaired *t* test: *t*(28) = 1.39; *P* = 0.175], and estrogen receptors *Esr1* (Mann-Whitney *U* test: *n* = 14 vs. 16; *U* = 90, *P* = 0.38) and *Esr2* (Mann-Whitney *U* test: *n* = 14 vs. 16; *U* = 68, *P* = 0.070). (**H**–**K**) Western blots of NAc core tissue showed no sex differences in tyrosine hydroxylase (Th) (Mann-Whitney *U* test: *n* = 8 vs. 15; *U* = 35, *P* = 0.12) (**I**), increased total DAT expression in male mice [unpaired *t* test: *t*(21) = 2.28, *P* = 0.033] (**J**), and increased phosphorylated DAT at the Thr53 phosphorylation site (p*Thr53* DAT) levels in female mice (Mann-Whitney *U* test: *n* = 8 vs. 15; *U* = 25, *P* = 0.024) (**K**). Note: the same actin image is reused in **I** and **J** for comparison. (**L**–**R**) FSCV in striatal slices measured dopamine release and clearance. (**M**) Example current versus time plot. (**N**) Electrically evoked dopamine release was enhanced in females [unpaired *t* test: *t*(32) = 2.120, *P* = 0.042]. (**O**) The V_max_ of dopamine in male and female mice [unpaired *t* test: *t*(29) = 1.65, *P* = 0.11]. (**P**–**R**) Sex comparisons of dopamine release and regulation including input–output curve [2-way repeated-measures ANOVA: no main effect of sex, *F*(1,19) = 1.45, *P* = 0.24; no sex × input interaction, *F*(3,57) = 1.864, *P* = 0.146] (**P**); increasing concentrations of calcium [main effect of sex, F(1, 12) = 5.52, *P* = 0.037; sex × calcium interaction, *F*(3, 33) = 7.20, *P* = 0.0008] (**Q**); and increasing concentrations of D2 receptor agonist quinpirole [2-way repeated measures ANOVA: no main effect of sex, *F*(1, 11) = 0.08, *P* = 0.78; no sex × drug interaction, *F*(3, 33) = 1.207, *P* = 0.32] (**R**). Data are presented as the mean ± SEM. *P < 0.05, **P < 0.01, ***P < 0.001. # indicates a significant interaction.

To assess stimulant effects on attentional performance, mice were trained in a modified psychomotor vigilance task in which responding to a brief visual cue was reinforced with sucrose (Figure 1A). Animals learned to withhold responding during the intertrial interval and to discriminate between illuminated and nonilluminated ports (Figure 1A, flowchart). Following acquisition (see Supplemental Figure 1 for baseline behavioral measures; supplemental material available online with this article; https://doi.org/10.1172/JCI178630DS1), we tested the effects of AMPH across a broad dose range (0.03–3.0 mg/kg) on performance, quantified as the percent change from baseline in hit rate (the proportion of correct responses) and in d′ (an index of discriminability between periods of reward availability and unavailability) (Figure 1, B and C). Across both measures, female mice exhibited greater sensitivity to AMPH than did male mice, with drug exposure producing a larger enhancement of hit rate and discriminability in females. Thus, the efficacy of stimulants on behavior in attentional tasks is enhanced in female mice.

### snRNA-Seq reveals sex-biased molecular signatures in core-projecting dopamine neurons.

To investigate whether sex differences in stimulant sensitivity reflect underlying molecular adaptations in dopamine circuits, we performed snRNA-Seq analysis of ventral tegmental area (VTA) dopamine neurons (35) and classified each neuron according to its downstream projection target, using marker gene-projection mapping strategies (Figure 1, D and E). Sox6/Calb1-expressing neurons (preferentially NAc core targeting) and Calb1/Aldh1a1-expressing neurons (preferentially shell targeted) were separated for analysis (Supplemental Figure 2). Core-projecting neurons displayed a larger number of sex-differentially expressed genes compared with shell-projecting neurons (Figure 1F), indicating that the NAc core is a site of pronounced molecular divergence between male and female mice. Within this core-projecting subpopulation, several genes central to dopamine synthesis and regulation—including tyrosine hydroxylase and DAT—showed sex differences in expression (Figure 1G). Furthermore, these neurons expressed estrogen receptors (Esr1 and Esr2) and estrogen receptor β (Esr2) expression trended toward increased expression in female mice. These projection-specific transcriptional differences along with the presence of estrogen receptors within this population highlight the NAc core as a potential substrate for sex-dependent modulation of dopamine function and psychostimulant sensitivity.

### Sex differences in DAT expression and phosphorylation.

VTA dopamine neurons send long-range projections to their target regions throughout the brain. Their cell bodies are localized in the midbrain, and thus gene expression analysis is done in cell bodies in VTA (Figure 1D). However, dopamine release and proteins regulating these functions are trafficked to distal axon release sites, such as the NAc core (Figure 1H). To this end, we next used Western blot analysis to examine protein expression in NAc core tissue punches (Figure 1H).

Consistent with gene expression data in this projection population, male mice showed a trend toward elevated tyrosine hydroxylase expression (Figure 1I) and higher levels of DAT compared with females (Figure 1J). However, although DAT levels were lower in female mice, the females exhibited increased phosphorylation of DAT at threonine 53 (Figure 1K, and see Supplemental Figure 3 for measurements that did not differ over the estrous cycle). DAT is dynamically regulated through cellular activity, trafficking, and posttranslational modifications, which can influence its localization and function (36). Notably, phosphorylation at Thr53 has been associated with altered transporter activity and heightened sensitivity to stimulants, independent of total protein expression (15, 28, 37). Thus, although female mice expressed lower overall DAT, they demonstrated higher levels of Thr53 phosphorylation, suggesting a potential mechanism by which transporter regulation contributes to sex-dependent differences in dopamine signaling and stimulant sensitivity.

### Sex-dependent regulation of dopamine dynamics.

**Figure 2:**
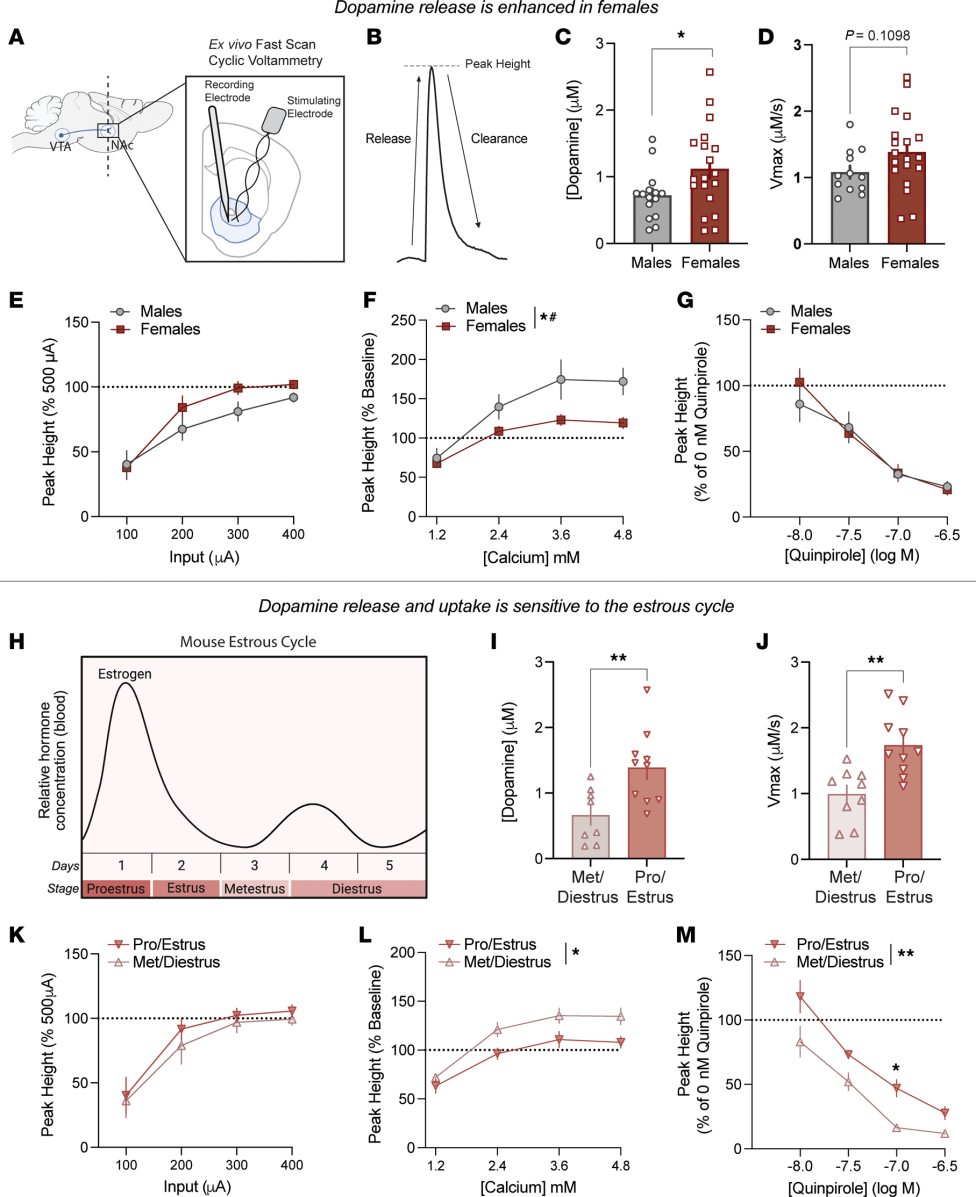
Dopamine release and its molecular regulation are modulated by biological sex and ovarian hormones. (**A**–**G**) FSCV in striatal slices measured dopamine release and clearance. (**B**) Example current versus time plot. (**C**) Electrically evoked dopamine release was enhanced in female mice [unpaired *t* test: *t*(32) = 2.120, *P* = 0.042]. (**D**) The V_max_ of dopamine uptake in male and female mice [unpaired *t* test: *t*(29) = 1.65, *P* = 0.11]. (**E–G**) Sex comparisons of dopamine release and regulation including input–output curve [2-way repeated measures ANOVA: no main effect of sex, *F*(1,19) = 1.45, *P* = 0.24; no sex × input interaction, *F*(3,57) = 1.864, *P* = 0.146] (**E**); increasing concentrations of calcium [main effect of sex, *F*(1, 12) = 5.52, *P* = 0.037; sex × calcium interaction, *F*(3, 33) = 7.20, *P* = 0.0008] (**F**); and increasing concentrations of the D2 receptor agonist quinpirole [2-way repeated measures ANOVA: no main effect of sex, *F*(1, 11) = 0.08, *P* = 0.78; no sex × drug interaction, *F*(3, 33) = 1.207, *P* = 0.32] (**G**). (**H**) Diagram of estradiol fluctuation across the mouse estrous cycle. Female mice were categorized into high-hormone (pro/estrus) or low-hormone (met/diestrus) states. (**I**) Dopamine release was enhanced in females in pro/estrous as compared with met/diestrus [unpaired *t* test: *t*(16) = 3.024, *P* = 0.0081]. (**J**) The V_max_ of dopamine uptake was enhanced in pro/estrus female mice compared with met/diestrus females [unpaired *t* test: *t*(17) = 3.67, *P* = 0.0019]. (**K**–**M**) Cycle-stage comparisons of dopamine release and key regulatory processes including input–output curve [2-way repeated measures ANOVA: no main effect of cycle stage, *F*(1,10) = 0.40, *P* = 0.54; no cycle stage × input interaction, *F*(3, 30) = 0.12, *P* = 0.95] (**K**); calcium sensitivity [main effect of cycle stage, *F*(1, 6) = 6.43, *P* = 0.044; no calcium × cycle stage interaction, *F*(3, 17) = 2.85, *P* = 0.068] (**L**); and response to increasing concentrations of D2 receptor agonist quinpirole [2-way, repeated-measures ANOVA: main effect of cycle stage, *F*(1, 7) = 23.40, *P* = 0.0019; no cycle stage × drug interaction, *F*(3, 21) = 0.66, *P* = 0.59] (**M**). Data are presented as the mean ± SEM. *P < 0.05, ***P < 0.001.

To measure dopamine release and clearance in live tissue, we conducted fast-scan cyclic voltammetry (FSCV) in coronal brain sections containing the NAc core. Dopamine release was evoked via an electrical pulse (500 μA) and recorded via a carbon-fiber recording electrode, allowing for dopamine measurements with subsecond resolution (Figure 2A). Because of the temporal and spatial specificity of this approach, dopamine release and clearance can be assessed independently of one another. Previous work has shown that, via FSCV, the decay of the signal (rate of clearance) is mediated via active transport through DAT (Figure 2B). Dopamine release was enhanced in female as compared with male mice (Figure 2C). There was a trend toward an increase in the maximal rate of dopamine reuptake (V_max_) in female mice relative to male mice (Figure 2D).

Next, we conducted a series of mechanistic studies pinpointing how molecular regulators of release contribute to these effects (Figure 2, E–G). Although stimulus-evoked dopamine release was increased in female mice at baseline (Figure 2C), this was not a function of differential sensitivity to stimulation intensity, which was the same between groups when plotted as a percent change from baseline (Figure 2E). Female mice were less sensitive to changes in calcium concentration than were their male counterparts (Figure 2F). This lack of ability of calcium to further increase release in female mice likely reflects the fact that evoked dopamine release is maximal in females and is thus less sensitive to effectors that typically further enhance release. Lastly, the D2 receptor agonist quinpirole decreased striatal dopamine release similarly in both sexes, with no significant sex differences (Figure 2G). Together, these data show that female mice have enhanced dopamine release and differences in DAT that likely change how they respond to stimulant medications.

### Enhanced dopamine release and clearance in female mice are modulated by the estrous cycle.

Given that dopamine neurons projecting to the core expressed estrogen receptors (Esr1 and Esr2) (Figure 1G) and that there is local estrogen receptor expression with the NAc itself, this neuronal population is positioned to be sensitive to circulating ovarian hormones, such as estradiol. Estradiol is known to fluctuate over the estrous cycle in female mice (Figure 2H). The estrous cycle phase was assessed by vaginal cytology immediately before sample preparation, allowing for the data to be parsed by phase at the time of recording, and female mice were categorized as either proestrus and estrus when estradiol levels were highest (combined and termed hereafter as pro/estrus), or metestrus and diestrus when estradiol levels are low and comparable to those of male mice (combined and termed hereafter as met/diestrus) (Figure 2H). Indeed, when female mice were separated by cycle stage, there was an increase in dopamine release (Figure 2I) and V_max_ (Figure 2J) in the pro/estrus phase as compared with the met/diestrus phase.

The molecular regulation of dopamine signaling also varied across the female cycle. Although neuronal excitability remained consistent across the cycle stage as assessed by input-output curve (Figure 2K), sensitivity to extracellular calcium was reduced during pro/estrus (Figure 2L). Similarly, D2-receptor–mediated inhibition of dopamine release by quinpirole was reduced in pro/estrus mice compared with met/diestrus mice (Figure 2M). These findings suggest that during pro/estrus, female mice exhibit a heightened dopamine response that is less responsive to typical modulatory mechanisms and serves as a substrate upon which AMPH and other stimulants act to enhance its effects on behavior.

### The pharmacodynamic effects of AMPH are modulated by the estrous cycle.

**Figure 3:**
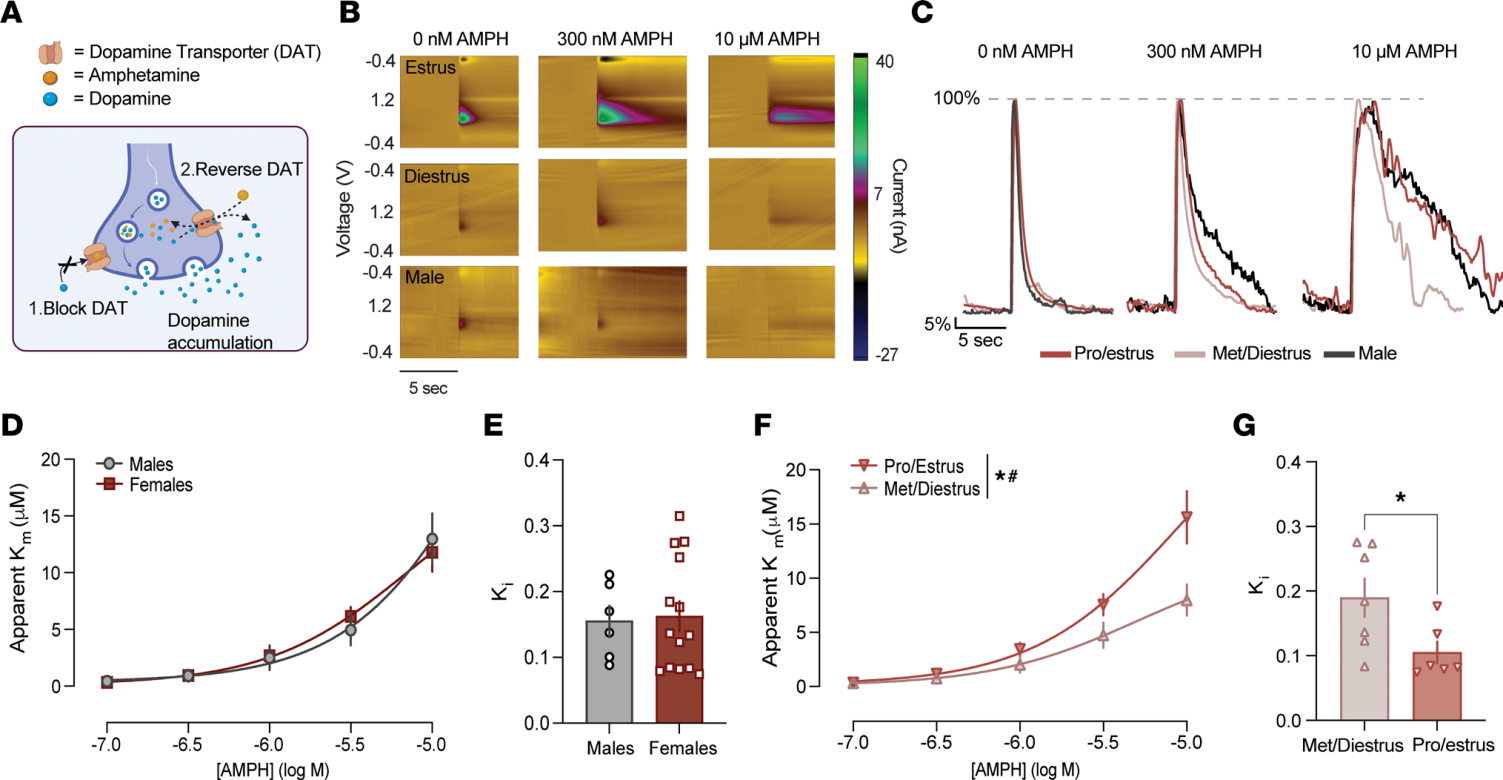
The pharmacodynamic effects of AMPH are modulated by the estrous cycle. (**A**) Diagram of AMPH’s mechanism of action. AMPH (yellow) acts through 2 primary mechanisms: (1) as a competitive uptake inhibitor, it blocks dopamine clearance at the DAT (orange); and (2) it actively induces dopamine efflux into the synaptic space by reversing transport through DAT. (**B**) Color plots showing dopamine (green; at its oxidation potential) at increasing concentrations of AMPH in female mice in estrus (top), diestrus (middle), and males (bottom). (**C**) Current versus time plots from each group. Signals are normalized to the peak height of each trace to highlight differences in clearance; release differences are not reflected, due to this normalization. (**D**) Cumulative concentration–response curves for AMPH (100 nM to 10 μM) in male and female mice. Apparent *K_m_* represents the extent to which AMPH inhibits dopamine clearance through the DAT. AMPH effects on clearance increased with concentration, with no significant sex differences [mixed-effects analysis: main effect of drug, *F*(1.332, 24.24) = 55.10, *P* < 0.0001; no main effect of sex, *F*(1, 19) = 0.0014, *P* = 0.97; no drug × sex interaction, *F*(5, 91) = 0.35, *P* = 0.88]. (**E**) *K_i_*, a measure of potency (the concentration required to produce 50% inhibition), for AMPH in male and female mice [unpaired *t* test: *t*(18) = 0.19, *P* = 0.86]. (**F**) Cumulative concentration–response curves for AMPH in females in pro/estrus versus met/diestrus, showing enhanced AMPH effects in proestrus [mixed-effects model: main effect of drug, *F*(1.426, 17.97) = 60.17, *P* < 0.0001; main effect of cycle stage, *F*(1, 13) = 6.79, *P* = 0.0218; drug × cycle stage interaction, *F*(5, 63) = 6.201, *P* < 0.0001]. (**G**) *K_i_* for AMPH in female mice in pro/estrus versus met/diestrus [unpaired *t* test: *t*(11) = 2.38, *P* = 0.037]. Data are presented as the mean ± SEM. *P < 0.05. # indicates an interaction.

AMPH is classified as a releaser and has 2 primary mechanisms of action (17–19). It prevents dopamine clearance by functioning as a competitive uptake inhibitor of DAT and also induces efflux of dopamine into the synaptic space by altering internal ionic gradients and thus reversing dopamine transport through both vesicular transporters and DAT (Figure 3A). As expected, increasing concentrations of AMPH produced a dose-dependent increase in extracellular dopamine levels. This effect was associated with a slower rate of dopamine clearance from the synaptic space, consistent with AMPH-induced inhibition of the DAT (Figure 3, B and C). Apparent *K_m_*, a measure of DAT inhibition derived from dopamine clearance kinetics, increased proportionally with AMPH concentration (Figure 3D). Elevated apparent *K_m_* values indicate reduced dopamine clearance efficiency, reflecting greater DAT blockade and an enhanced pharmacological effect of AMPH. Additionally, the *K_i_*, defined as the concentration of AMPH required to produce uptake inhibition at 50% of maximal inhibition, indicates drug potency (as a concentration) (Figure 3E).

Although there was no difference between male and female mice in AMPH potency as measured by apparent *K_m_* (Figure 3D) or *K_i_* (Figure 3E), there were robust effects of cycle stage on these measures in females. AMPH potency was enhanced in female mice in pro/estrus compared with met/diestrus as measured by apparent *K_m_* (Figure 3F) and *K_i_* (Figure 3G).

To parse the effects of AMPH on release (independent of measures of clearance), we assessed the peak of dopamine evoked by different stimulation parameters (Supplemental Figure 4). Over increasing concentrations, AMPH progressively reduced dopamine release evoked by stimulations mimicking the tonic (baseline) firing of dopamine neurons (38). However, high concentrations of AMPH increased dopamine evoked by phasic stimulations (which occur in vivo in response to salient stimuli). This was a unique feature of AMPH that was not seen with methylphenidate (Supplemental Figure 5). These measures were not different across sex or hormone stage for either drug. Collectively, these findings indicate that AMPH’s effects on the dopamine system in female mice are strongly modulated by DAT-dependent mechanisms and by fluctuations in ovarian hormones, both of which act to enhance drug potency.

### The pharmacodynamic effects of methylphenidate are enhanced in female mice.

**Figure 4:**
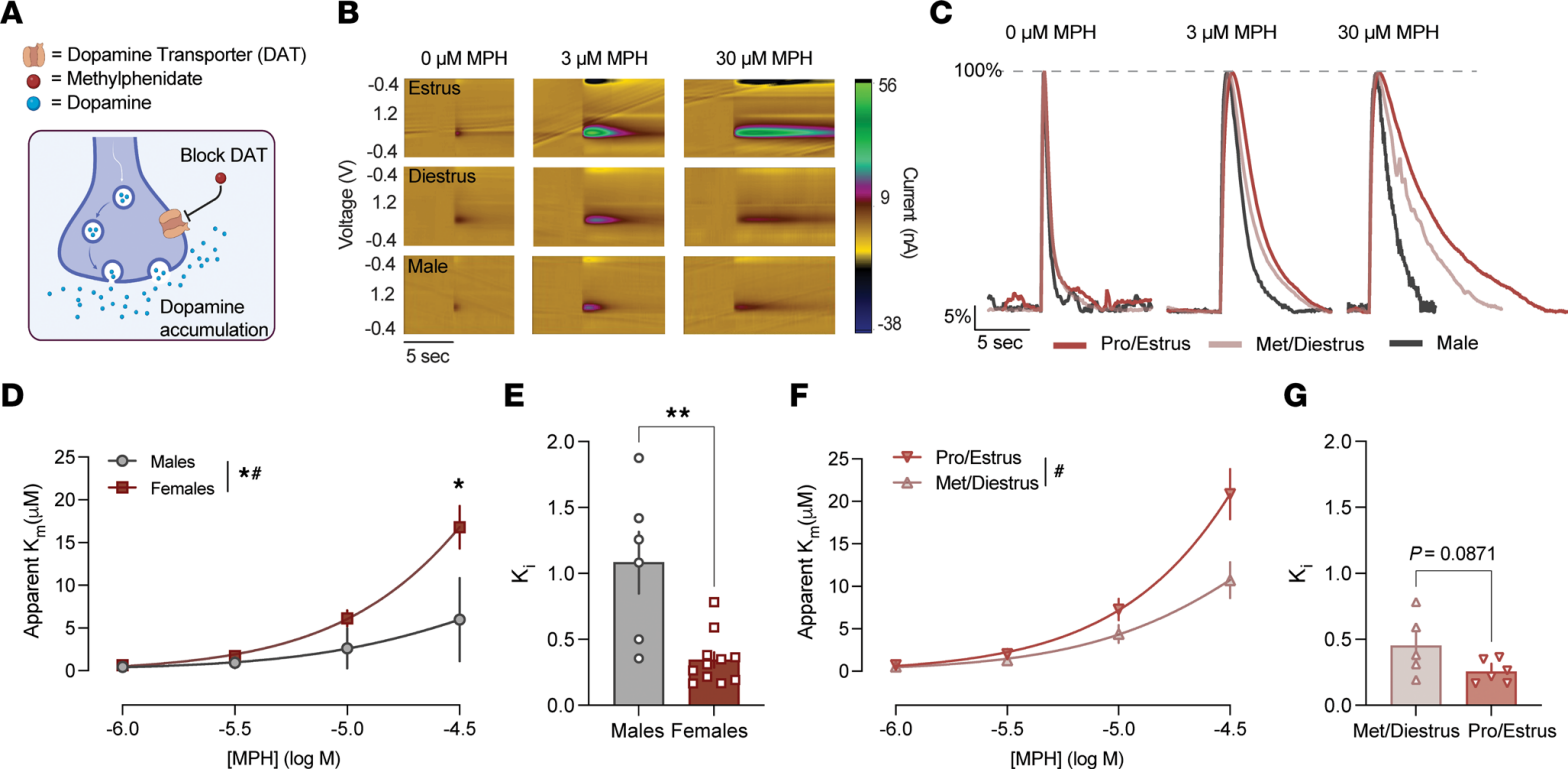
The pharmacodynamic effects of methylphenidate are enhanced in females and sensitive to estrous cycle. (**A**) Diagram of methylphenidate’s mechanism of action. Methylphenidate (red) blocks the DAT (orange). (**B**) Color plots showing dopamine (green; at its oxidation potential) over increasing concentrations of methylphenidate in females in estrus (top), diestrus (middle), and males (bottom). (**C**) Current versus time plots from each group. Signals are normalized to the peak height of each trace to highlight differences in clearance; release differences are not reflected due to this normalization. (**D**) Cumulative concentration–response curves for methylphenidate (1–30 μM). Methylphenidate’s effects on dopamine clearance increased with concentration and were enhanced in female mice relative to males [mixed-effects analysis: main effect of drug, *F*(1.060, 15.20) = 40.74, *P* < 0.0001; main effect of sex, *F*(1, 15) = 8.42, *P* = 0.0110; drug × sex interaction, *F*(3, 43) = 10.03, *P* < 0.0001]. (**E**) *K_i_* for methylphenidate in male and female mice. *K_i_* was decreased in females, indicating increased potency [unpaired *t* test: *t*(14) = 3.72, *P* = 0.0023]. (**F**) Cumulative concentration–response curves for methylphenidate in females in pro/estrus versus met/diestrus [2-way repeated-measures ANOVA: main effect of drug, *F*(1.103, 8.83) = 60.21, *P* < 0.0001; no main effect of cycle stage, *F*(1, 8) = 4.19, *P* = 0.075; dose × cycle stage interaction, *F*(4, 32) = 6.402, *P* = 0.0007]. (**G**) *K_i_* of methylphenidate during pro/estrus and met/diestrus [unpaired *t* test: *t*(9) = 1.92, *P* = 0.087]. Data are presented as the mean ± SEM. *P < 0.05, **P < 0.01. # indicates an interaction.

We were interested in understanding if the hormone-sensitive properties of AMPH applied to other prescribed psychostimulants that differ in their pharmacodynamic actions. Methylphenidate is classified as a reuptake inhibitor and acts by inhibiting DAT function, leading to an accumulation of dopamine in the synaptic space (13) (Figure A). Predictably, methylphenidate effects on dopamine increased as the concentration of methylphenidate increased, indicating decreased clearance of dopamine from the synaptic space in the presence of methylphenidate (Figure 4, B and C). The extent to which methylphenidate inhibited dopamine clearance through DAT was enhanced in female mice compared with males, indicated by an increased apparent *K_m_* in females. (Figure 4D). There were no effects of methylphenidate on any measures of release (Supplemental Figure 5). Additionally, *K_i_* was decreased in females, indicating an increase in potency (i.e., less methylphenidate is needed to produce the same amount of DAT inhibition) (Figure 4E).

### Sex differences in methylphenidate potency are sensitive to, but not dependent on, the estrous cycle.

When female mice were separated by cycle stage, the extent to which methylphenidate inhibited dopamine clearance through DAT was enhanced in female mice in pro/estrus compared with met/diestrus (Figure 4F). Similarly, there was a trend toward a reduction of the *K_i_* of methylphenidate in pro/estrus as compared with met/diestrus, indicating that methylphenidate likely had a higher potency during phases when hormone levels are elevated (Figure 4G). Together, these data show that the effects of methylphenidate on dopamine clearance through DAT are greater in female mice regardless of cycle stage, and the estrous cycle stage does contribute to some of these effects.

### Ovarian hormones play a central role in sex differences in baseline dopamine dynamics.

**Figure 5:**
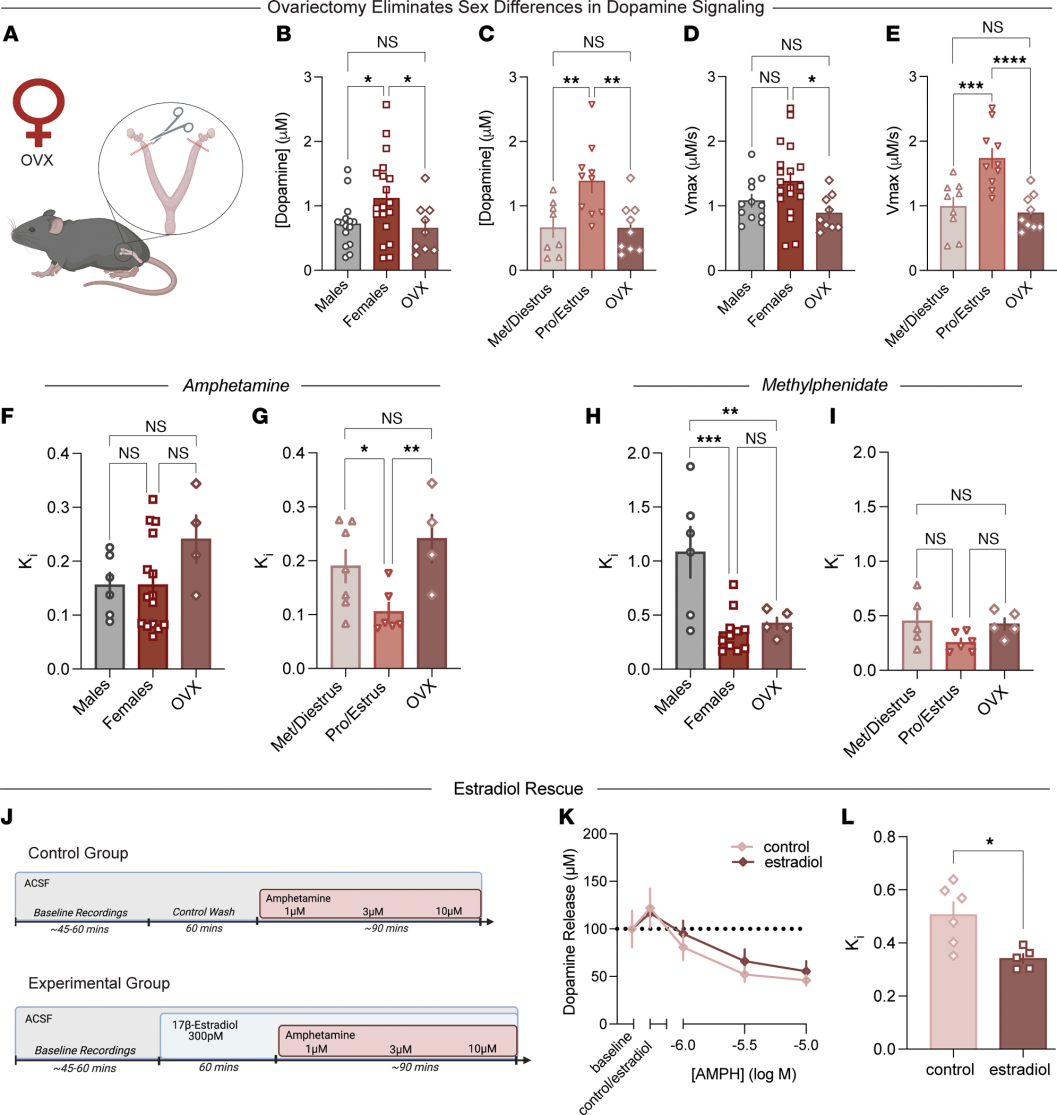
Ovarian hormones are required for sex differences in basal dopamine function and AMPH sensitivity. (**A**–**G**) Voltammetry recordings were repeated in OVX females. Data show that ovariectomy eliminated sex differences in dopamine release and drug effects. (**B**) Peak dopamine release was compared among OVX females, freely cycling females, and males [1-way ANOVA: *F*(2, 40) = 3.58, *P* = 0.037]. (**C**) Dopamine release was further compared across OVX females and cycling females in met/diestrus and pro/estrus [1-way ANOVA: *F*(2, 24) = 7.49, *P* = 0.003]. (**D**) Maximal rate of reuptake was assessed in OVX females and compared with males and freely cycling females [1-way ANOVA: *F*(2, 37) = 3.95, *P* = 0.028]. (**E**) Reuptake rates were also compared across OVX females and cycling females in metestrus/diestrus and proestrus/estrus [1-way ANOVA: *F*(2, 25) = 13.07, *P* = 0.0001]. (**F** and **G**) OVX eliminated sex differences and effects of estrous cycle in females. (**F**) *K_i_* for AMPH in OVX females was assessed and compared with males and freely cycling females [1-way ANOVA: *F*(2, 22) = 1.87, *P* = 0.177]. (**G**) *K_i_* for AMPH was also assessed across cycle stage in met/diestrus and pro/estrus females [1-way ANOVA: *F*(2, 14) = 4.89, *P* = 0.025]. (**H** and **I**) OVX did not eliminate the enhanced potency of methylphenidate in females compared with males. (**H**) *K_i_* for methylphenidate in OVX females was measured and compared with males and freely cycling females [1-way ANOVA: *F*(2, 19) = 10.34, *P* = 0.0009]. (**I**) *K_i_* for methylphenidate was further assessed across cycle stage in cycling females [1-way ANOVA: *F*(2, 13) = 2.67, *P* = 0.11]. (**J**–**L**) Experiments were run to determine whether local 17β-estradiol in the NAc core could rescue OVX-induced reductions in AMPH potency. (**J**) Schematic of ex vivo voltammetry recordings in OVX NAc slices. We performed ex vivo voltammetry and bath-applied 17β-estradiol to slices containing the NAc before running an AMPH concentration response curve. (**K**) Cumulative concentration–response curve of dopamine release for AMPH in control versus estradiol-rescue groups, showing no effects on the dopamine release component of AMPH actions. (**L**) *K_i_* for AMPH, which is determined by the ability to inhibit the DAT, in OVX females in control versus estradiol-rescue groups showing no difference in AMPH effects on dopamine release [unpaired *t* test: *t*(9) = 3.06, *P* = 0.014]. Data are presented as the mean ± SEM. *P < 0.05, **P < 0.01, ****P < 0.0001.

The data we have reported thus far suggest that ovarian hormones play a central role in sex differences in dopaminergic control of behavior at baseline as well as in response to stimulant medications. To casually link hormonal state to these effects, we repeated a range of experiments in ovariectomized (OVX) female mice (Figure 5A). Indeed, ovariectomy eliminated sex differences in release and uptake measures at baseline. Dopamine release in OVX female mice was reduced, as compared with intact females, to levels comparable with male mice (Figure 5B). Furthermore, ovariectomy resulted in dopamine release that was lower as compared with intact female mice in pro/estrus and comparable to females in met/diestrus (Figure 5C). The same trend was observed with measures of dopamine reuptake. Ovariectomy resulted in a reduction in V_max_ as compared with intact female mice (Figure 5D). Furthermore, ovariectomy reduced V_max_ as compared with intact female mice in pro/estrus to levels comparable to female mice in met/diestrus (Figure 5E). These findings demonstrate that elevated dopamine system function observed in intact female mice is largely driven by the presence of circulating hormones.

### Ovarian hormones are necessary for hormone-sensitive effects of AMPH, but not methylphenidate effects.

Next, we defined how ovariectomy influenced AMPH and methylphenidate potency. Although AMPH potency was comparable across male, intact female, and OVX female groups (Figure 5F), when the data were parsed by cycle stage, ovariectomy reduced AMPH potency, with a higher *K_i_* in OVX female mice as compared with the intact proestrus group (Figure 5G). Conversely, methylphenidate (MPH) effects were unaffected by ovariectomy. The *K_i_* of MPH in OVX female mice was no different than in intact females (Figure 5H), and there was no significant effect when compared across the estrous cycle (Figure 5I).

### Local estradiol replacement in the NAc core rescues AMPH potency.

Given AMPH’s unique sensitivity to hormonal fluctuations, we hypothesized that we could reverse ovariectomy-induced reductions in AMPH potency using ex vivo estradiol application locally. We bath-applied 17β-estradiol on brain slices from OVX female mice for 1 hour and then constructed an AMPH concentration response curve (Figure 5J). Estradiol application did not affect AMPH effects on dopamine release (i.e., the peak amount of dopamine evoked in the presence of AMPH) (Figure 5K), but it did increase AMPH potency, as reflected by a reduced *K_i_* (Figure 5L). Together, these data show that AMPH effects on the dopamine system are highly hormone sensitive, leading to enhanced behavioral effects in attention-related tasks, likely through local actions of ovarian hormones in reward-related brain regions, such as the NAc core.

## Discussion

We provide comprehensive evidence that biological sex and ovarian hormone cycles are key regulators that influence stimulant effects through multiple, complementary mechanisms. Our findings span behavior to molecular substrates, revealing that sex differences in stimulant pharmacodynamics emerge from enhanced dopamine system function in female mice that is dynamically regulated by ovarian hormones. Importantly, we demonstrate that AMPH and methylphenidate, despite both increasing synaptic dopamine levels, have distinct patterns of sex and hormone sensitivity that reflect their different mechanisms of action. These data highlight that sex differences are not uniform but are dependent on a range of factors that include drug pharmacology, ovarian hormones, and the interaction between these factors.

Emerging clinical evidence suggests that females show enhanced sensitivity to stimulant medications, but the underlying mechanisms remain unclear. Neuroimaging studies have reported sex differences in stimulant-induced dopamine system responses, including enhanced methylphenidate effects on dopamine transmission and glucose metabolism in females compared with males (10, 39), suggesting fundamental sex differences in how these drugs interact with dopamine circuitry. Indeed, here we show that female mice demonstrated enhanced sensitivity to stimulants in attention-related tasks and show that, under some conditions, both methylphenidate and AMPH have enhanced effects on dopamine levels in female mice.

The primary substrate for stimulant actions on the brain and behavior is the dopamine system. Dopamine neurons originate in the midbrain and send projections throughout the brain to regulate neural dynamics and behavior. We show here that dopamine projections from the VTA to the NAc core are a key hub mediating sexually dimorphic dopamine effects. Using snRNA-Seq from VTA dopamine neurons, we used genetic markers to identify putative NAc core versus NAc shell-projecting dopamine neurons. We show that dopamine projections to the NAc core represent a primary locus of sexually dimorphic gene expression. Core-projecting dopamine neurons showed more differentially expressed genes (DEGs) between sexes, including key regulators of dopamine synthesis (*Th*) and transport (*Slc6a3*). This finding is particularly relevant given that the NAc core is implicated in attention and cognitive processes targeted by stimulant medications. The presence of estrogen receptors (*Esr1*, *Esr2*) in these sexually dimorphic neurons, as well as locally in the microcircuits at their projection targets in the NAc, provides a molecular mechanism for hormone-sensitive modulation of stimulant effects.

Female mice exhibited enhanced dopamine system function at baseline that served as a substrate for increased stimulant sensitivity. Dopamine release was enhanced in female mice under all conditions, and reuptake through DAT was increased during proestrus, suggesting that the system is primed for release and efficient repackaging. Furthermore, pharmacology experiments to probe molecular substrates that contribute to release regulation demonstrate that evoked dopamine release in female mice was near maximal levels and showed reduced responsiveness to modulatory inputs. For example, diminished sensitivity to D2 autoreceptor inhibition in pro/estrus female mice indicated that hormonal states created conditions in which the dopamine system is primed for maximal responses but less constrained by negative feedback mechanisms. At the protein level, whereas male mice had higher total DAT expression, females had elevated phosphorylated DAT, which is associated with enhanced activity, explaining how enhanced transporter-mediated clearance occurs despite lower total transporter levels. Importantly, this phosphorylation site has been specifically linked to enhanced effects of some psychostimulants at the DAT (15, 28, 37). Together, these findings reveal that female mice possess an inherently more robust and less regulated dopamine system that creates optimal conditions for enhanced stimulant effects across multiple pharmacological mechanisms.

Ovariectomy studies provide direct evidence that ovarian hormones mediate, at least in part, sex differences in stimulant responses. Among the multiple hormones that fluctuate during ovarian cycles (including estrogens, progestins, follicle-stimulating hormone, and luteinizing hormone), estrogens exert the most robust effects on dopamine system function, increasing dopamine neuron firing rates, enhancing dopamine release, and elevating extrasynaptic dopamine levels (28, 33, 40–42). Previous studies support estradiol’s primacy in modulating stimulant effects. Exogenous estradiol administration increases cocaine and AMPH effects on striatal dopamine levels (24, 43–45); females in estrus exhibit enhanced cocaine effects on the DAT compared with both diestrus females and males (28). Our findings directly extend this work by demonstrating that AMPH potency is reduced in OVX female mice and could be rescued by ex vivo estradiol application, indicating that local hormone action in the NAc is sufficient to restore enhanced drug sensitivity. This mechanistic finding aligns with human studies reporting greater sensitivity to AMPH-based medications during the follicular phase when estradiol levels are elevated (11, 12). In contrast, although methylphenidate’s effects varied across estrous cycle stages, its potency was comparable between intact and OVX female mice, indicating that circulating ovarian hormones were not required for the enhanced drug potency observed in female mice relative to males. This divergence suggests AMPH’s active release mechanisms interact more strongly with hormone-sensitive aspects of dopamine system function, highlighting how different pharmacological mechanisms can exhibit distinct patterns of hormonal regulation.

These findings provide mechanistic insight into observed sex differences in stimulant pharmacodynamics that may differ across drug classes. The estradiol-sensitive enhancement of AMPH effects offers mechanistic context for clinical observations of menstrual cycle–related variations in stimulant responses. This is an important consideration because this could affect both the efficacy and side effect profiles of this medication specifically in women. Indeed, women report increased side effects of these stimulant medications (8). Given the widespread use of hormonal contraceptives, it also is possible that contraceptive drugs may change the efficacy of these drugs and ultimately change the effective dosage in women (46, 47). Furthermore, enhanced dopamine responses in females, particularly during high-estradiol phases, could contribute to faster progression from use to addiction, greater difficulty with abstinence, and enhanced craving responses observed clinically (48–50).

Together, this work demonstrates that sex differences in stimulant pharmacodynamics arise from multiple mechanisms spanning molecular, cellular, and systems levels. The enhanced dopamine system function in females, dynamically regulated by ovarian hormones, creates conditions for increased stimulant sensitivity that varies by drug mechanism. Although men have historically been diagnosed with ADHD and treated with stimulants at higher rates (51–53), women are now closing that gap (54, 55). This presents a critical lack of understanding of how these medications may adversely affect women. Understanding these fundamental biological differences becomes increasingly critical for optimizing treatment efficacy while minimizing adverse effects.

## Methods

### Sex as a biological variable

Both male and female animals were included in this study, and sex was considered a biological variable in study design and data analysis. Analyses were performed by sex where applicable. Experiments examining the influence of ovarian hormones were conducted in female animals only using ovariectomy and estradiol-replacement paradigms, as described later in Methods.

### Mice

Male and female 8-week-old C57BL/6J mice (*n* = 133) were obtained from The Jackson Laboratory (SN: 000664). All animals were kept under a 12-hour reverse light/dark cycle with unlimited access to food and water.

### Drug information

AMPH and methylphenidate were generously provided by the National Institute on Drug Abuse Drug Supply program.

### Behavior

#### Mice.

A total of 23 adult C57BL/6J mice (*n* = 12 males and 11 females) were used. Mice were housed in groups of 3–4/cage under a 12-hour reverse light/dark cycle with ad libitum access to water. To motivate task performance, mice were food restricted beginning approximately 5 days prior to training. Each mouse received 3.2 g food pellets per day, provided after the behavioral session. For female mice, food was given following vaginal cytology assessment.

#### Psychomotor vigilance task.

A modified psychomotor vigilance task was used to assess sustained attention in mice (56). Behavioral testing was conducted in standard operant conditioning chambers equipped with 2 nose-poke ports (Med Associates). Each daily session lasted 60 minutes. A trial was initiated by illumination of the house light. After a variable intertrial interval (15, 30, or 45 seconds), 1 of the 2 nose-poke ports, randomly selected, was illuminated (*P* = 0.5 for either port on a given trial). Mice were required to nose-poke the illuminated port within 5 seconds to receive a reinforcer (a 20% sucrose solution), which was delivered at a spout located between the 2 ports. Correct responses resulted in the house light turning off and delivery of sucrose. After 10 seconds, re-illumination of the house light signaled the start of a new trial.

Failure to respond within the 5-second window was recorded as a miss, after which the house light was extinguished for a 10-second time-out period before the start of a new trial. Incorrect responses (i.e., a nose-poke in the nonilluminated port) and premature responses (i.e., a nose-poke when neither port was illuminated) also triggered a 10-second time-out and initiation of a new trial.

During initial training, mice were given 10 seconds to respond, and this criterion was gradually reduced to 5 seconds based on individual performance. Only sessions in which mice responded within the 5-second window were used for analysis.

#### Drug administration.

Following acquisition of stable baseline performance for at least 3 consecutive days (with <30% variability in hit rate), mice underwent pharmacological testing. *d*-AMPH dissolved in 0.9% saline was administered intraperitoneally at doses of 0.01, 0.03, 0.3, 1, and 3 mg/kg in a pseudorandomized order. On drug-testing days, mice were weighed and injected immediately before placement in the operant chamber. Sessions began 10 minutes after injection. Saline injections were given on control days. Full dose–response function was replicated in all mice, and data reflect a mean of both determinations.

#### Data collection and analysis.

Behavioral performance was initially quantified as the proportion of trials that result in a correct response (hits), omission (misses), or premature response (false alarms) across a 1-hour session. Because obvious sex differences were observed (Supplemental Figure 1), the effects of *d*-AMPH were assessed as percent change from baseline measures. Baseline performance for each mouse was derived from the last 3 training sessions before each drug testing regimen. The dose response function for *d*-AMPH was determined by the average of the 2 separate drug-testing cycles.

In addition to basic performance measures, a d′ index of discriminability was calculated for each session using the following equation: d′ = z(hit rate) – z(false alarm rate).

High discriminability indices are the product of a high rate of correct responses in conjunction with a low number of false alarms. This measure provides a discrete value that describes a mouse’s ability to differentiate between periods of reward availability (cue on) from unavailability (cue off). Deficits in discriminability conventionally reflect attentional deficiencies and can reveal nonspecific motoric responses that may be induced by drug treatment.

### snRNA-seq

#### Preprocessing and clustering.

snRNA-seq data from 2 female and 2 male samples of rat VTA tissue (GSE168156) were processed using Scanpy and single-cell variational inference (scVI) (35, 57, 58). Raw count matrices were preprocessed individually before integration. Cells were retained if they expressed at least 200 genes, and genes were retained if they were expressed in at least 3 cells. Cells exceeding the 98th percentile of total genes expressed or having mitochondrial gene expression greater than 5% were removed. Doublet detection and removal were performed using Scrublet with default parameters (59). Raw counts were normalized to a target sum of 1,000,000 counts per cell before log-transformation (normalized log1p values). Highly variable genes were identified using a batch-aware approach and the top 6,000 genes across samples were selected; *Drd2*, *Calb1*, *Sox6*, *Slc17a6*, *Vip*, and *Aldh1a1* were manually included regardless of variability.

scVI was used for data integration, using sample identity as a batch key and incorporating mitochondrial percentage and total counts as continuous covariates. The scVI latent representation was used for uniform manifold approximation and projection embedding (default: 15 nearest neighbors), followed by Leiden clustering at 0.25 resolution. The Leiden cluster corresponding to dopamine neurons was identified according to expression of the neuronal marker gene *Syt1* and the dopamine neuron marker genes *Th* and *Slc6a3*.

#### Identification of shell-projecting and core-projecting neurons.

We used the mapping between marker gene and projection target described by Poulin et al. (60) to define NAc core- and shell-projecting neurons. Core-projecting neurons were identified as nuclei that expressed both Calb1 and Sox6 (*n* = 16 females and *n* = 14 males); shell-projecting neurons were identified as nuclei that expressed both Calb1 and Aldh1a1 (*n* = 24 females and *n* = 34 males) (Supplemental Figure 1A).

#### Gene expression analyses.

The number of DEGs between core and shell regions was evaluated. To account for differences in power when computing the number of DEGs between male and female mice within each subgroup, we performed repeated random sampling (*n* = 50 bootstrap samples per region), selecting with replacement 30 nuclei per sex in each iteration to ensure balanced comparisons. For each bootstrap, samples were concatenated and Wilcoxon rank-sum tests were applied using Scanpy’s rank_genes_groups function on normalized log1p values to identify DEGs between sexes. DEGs were filtered to include only genes with an absolute log-fold change greater than 0.5 and a Benjamini-Hochberg adjusted *P* value of less than 0.025 (Bonferroni correction for 2 regions). A Mann-Whitney *U* test was conducted to statistically compare the number of DEGs detected between core- and shell-projecting neurons.

#### Sex-dependent gene expression in core-projecting neurons.

Expression of genes of interest (*Th*, *Slc6a3*, *Maoa*, *Esr1*, *Drd2*, *Esr2*, *Slc18a2*) between male and female mice in core-projecting neuronal nuclei (*n* = 16 female and 14 male) were performed on scVI-normalized expression values, which represent denoised, batch-corrected estimates of transcript abundances. For each gene pair, data that met assumptions of normality and equal variance were analyzed using an unpaired *t* test; otherwise, a Mann–Whitney test was used.

### Western blot analysis of DAT expression in striatal tissue

#### Tissue collection.

A total of 18 mice (*n* = 6 males and *n* = 12 females) were rapidly decapitated. Their brains were dissected and submerged in oxygenated, sucrose-supplemented artificial cerebrospinal fluid (aCSF). Bilateral NAc core tissue punches were obtained using a 1,200 mm diameter tissue puncher (69033-12, Electron Microscopy Sciences) from 300 μm thick coronal brain sections. Samples were then immersed in oxygenated aCSF.

#### Generation of NAc core whole-cell lysates.

Tissue punches were mechanically homogenized using a mortar and pestle at 4°C in 300 μL of RIPA lysis buffer (MilliporeSigma, R0278; 150 mM NaCl, 1.0% IGEPAL CA-630, 0.5% sodium deoxycholate, 0.1% SDS, 50 mM Tris, pH 8.0) supplemented with protease and phosphatase inhibitors (Thermo Fisher Scientific, 78446). After lysis, samples were centrifuged at 10,000*g* for 5 minutes at 4°C. After centrifugation, the supernatant was transferred to a new prechilled tube. The concentration of protein in each sample was quantified using the Qubit Protein Assay Kit (Invitrogen, Thermo Fisher Scientific, Q33212) on an Qubit 4 Fluorometer (Invitrogen, Thermo Fisher Scientific, Q33238).

#### SDS-PAGE.

We mixed 6 μg protein in RIPA buffer with 4× Laemmli Sample Buffer (Bio-Rad, 1610747; 3 parts lysate, 1 part buffer) supplemented with 10% 2-ME and boiled at 95°C for 5 minutes. Samples were loaded onto 4%–20% precast polyacrylamide gels (Bio-Rad, 5671094) and proteins separated by electrophoresis.

#### Western blotting.

Proteins were transferred onto 0.45 μm pore PVDF membranes (Thermo Fisher Scientific B88585) at 100 volts for 30 minutes at 4°C in SDS-free transfer buffer containing 20% methanol. Membranes were blocked in Tris-buffered saline containing 5% BSA and 0.1% Tween-20 for 2 hours at room temperature. Membranes were incubated in primary antibody in blocking solution for 12 hours at 4°C (anti–β actin antibody; Abcam, ab8227; 1:1,000), anti-DAT antibody (MilliporeSigma, AB2231; 1:500), anti–tyrosine hydroxylase antibody (MilliporeSigma, AB152; 1:500). Membranes were washed in Tris-buffered saline containing 0.1% Tween-20 and incubated with a goat anti–rabbit IgG HRP-conjugated secondary antibody (Promega, PR-W4011; 1:5,000) and Precision Protein StrepTactin-HRP Conjugate (Bio-Rad, 1610381; 1:10,000) in blocking solution for 5 hours at 4°C. Bands were quantified by densitometry using Fiji software (NIH). Density values were normalized to β-actin loading control or for posttranslational modifications to the nonmodified protein (Supplemental Figure 6).

### FSCV

#### Data collection.

Ex vivo FSCV was used to characterize dopamine release and uptake dynamics in the NAc. This was done both at baseline and in the presence of AMPH or methylphenidate. Animals were consistently sacrificed during their dark cycle, the phase of the circadian rhythm associated with peak dopamine release (61). A vibrating tissue slicer was used to prepare 300 μm thick coronal brain sections containing the NAc core. The tissue was then immersed in oxygenated aCSF containing: 126 mM NaCl (126), 2.5 mM KCl (2.5), 1.2 mM NaH_2_PO_4_, 2.4 mM CaCl_2_, 1.2 mM MgCl_2_, 25 mM NaHCO_3_, 11 mM glucose, 0.4 mM l-ascorbic acid, and pH adjusted to 7.4. Slices were then transferred to the resting chambers containing aCSF at 32°C and a flow rate of 1 mL/min. A carbon-fiber microelectrode (100–200 μM length, 7 μM radius) and bipolar stimulating electrode were placed close to the NAc core. Extracellular dopamine was recorded by applying a triangular waveform (–0.4 to +1.2 to –0.4 V vs. Ag/AgCl, 400 V/s). For tonic stimulation, a single electrical pulse (350 μA, 4 ms, monophasic) was applied to the tissue every 3 minutes to evoke dopamine release until the signal stabilized (<10% variability in peak height of signal within 3 recordings). For phasic stimulation, dopamine release was evoked with 5 electrical pulses (500 μA, 4 ms, monophasic) at 5 Hz, 10 Hz, and 20 Hz. Each slice was used for a single experimental condition unless otherwise specified. Data were counterbalanced, and all groups were randomized over time. For each recording, *n* referred to the number of slices.

Recording electrodes were calibrated by measuring the responses in electrical current (nA) produced by a known concentration of dopamine (3 μM) using a flow injection system. This calibration process allowed for the conversion of electrical current readings to dopamine concentrations.

#### Input–output curve, calcium concentration curve, and quinpirole dose–response experiments.

Input–output curves were generated by incrementally increasing stimulation intensity, with peak amplitudes normalized to baseline values. For extracellular calcium experiments, slices were perfused with aCSF containing increasing Ca²^+^ concentrations (1.2, 2.4, 3.6, and 4.8 mM). Quinpirole dose–response experiments were conducted by bath application of cumulatively increasing concentrations of quinpirole.

#### AMPH and methylphenidate effects on DAT.

Slices were bath-applied with increasing concentrations of AMPH (100 nM, 300 nM, 1 μM, 3 μM, 10 μM) or methylphenidate (1 μM, 3 μM, 10 μM, 30 μM). Dopamine release was evoked at each concentration until stable responses were obtained and then the next concentration was applied. Data were analyzed (as described later in this section) to assess drug potency and effects on dopamine reuptake.

#### AMPH and methylphenidate effects on release.

Once dopamine release was stabilized at the highest concentration of each drug, tonic stimulations (*n* = 1 pulse) and phasic stimulations were applied (*n* = 5 pulses at 5–20 Hz) to assess frequency-dependent release dynamics in the presence of methylphenidate and AMPH. To assess the contribution of D2 autoreceptors to these effects, 1 μM and 10 μM of AMPH were bath-applied to slices, followed by coapplication with 1 μM sulpiride.

#### Estradiol experiments.

Following stabilization of dopamine release at baseline, 17β-estradiol (300 pM; experimental group) or aCSF (control group) was bath-applied to slices for 1 hour. AMPH was then applied in increasing concentrations (1, 3, and 10 μM), and evoked dopamine release was recorded at each concentration until stable responses were achieved. The amount of dopamine release and *K_i_* were determined as described.

#### Voltammetric data analysis.

For all analyses of FSCV data, Demon voltammetry and analysis software was used (62). Data were modeled via 2 approaches: (a) using Michaelis-Menten kinetics to determine dopamine release and V_max_ or (b) using peak and decay kinetics to determine the peak height of the dopamine signal. In the case of Michaelis-Menten modeling, all parameters were considered as floating, and the best fit line was determined for each data point (28). The parameter apparent *K_m_* was used as a measure of the ability of stimulants to inhibit dopamine clearance. Elevated apparent *K_m_* values indicate reduced dopamine clearance efficiency, reflecting greater DAT blockade and an enhanced pharmacological effect of AMPH. *K_i_* values were determined by plotting the linear concentration effect profiles and determining the slope of the linear regression. The *K_i_* was calculated by the equation *K_m_*/slope, where *K_m_* is affinity of dopamine for the DAT at baseline. *K_i_* values are reported in mM and are a measure of the drug concentration that is necessary to produce 50% uptake inhibition. *K_i_* is a measure of the drug affinity for the DAT, reported in concentration of drug, and apparent *K_m_* is a dopamine-related parameter that estimates the amount of dopamine uptake inhibition.

#### Vaginal cytology.

To monitor the naturally occurring estrous cycle of female mice, vaginal cytology was performed. The lavage technique (63) was performed before rapid decapitation to confirm that female mice were in either met/diestrus (low circulating hormone levels) or estrus (high circulating hormone levels).

### Statistics

The data were examined using GraphPad Prism, version 10.0 (GraphPad Software), or, for generation of probability density curves, the kernel density estimation function “seaborn” from the Python library (64). When comparing 2 groups, unpaired *t* tests were conducted, unless otherwise stated. In all other experiments, 2-way ANOVA was used, along with Holm−Šidák’s multiple comparisons tests, which included planned tests for making pairwise comparisons in cases where there was no interaction. For protein analysis, for each protein analyzed, data that met assumptions of normality and equal variance were analyzed using an unpaired *t* test; otherwise, a Mann-Whitney *U* test was used. Statistical outliers were determined based on each individual data set. The type 1 error rate (α) for all statistical tests was set to 0.05. Data in the figures are presented as the mean ± SEM.

### Study approval

All experiments were conducted in accordance with the IACUC at Vanderbilt University School of Medicine, which approved and supervised all animal protocols.

### Data availability

Values for all data points in graphs are reported in the Supporting Data Values file. The data that support the findings of this study are available from the corresponding author upon reasonable request. Raw data from the publicly available datasets analyzed in this study are available in the Gene Expression Omnibus (GEO) database (GEO GSE168156).

## Author contributions

JT, BAC, and ESC conceptualized and designed the study. BAC, JT, MZL, SDE, SR, AKP, HBR, HBE, DMG, LZ, HJY, SHL, ALC, LJB, EBH, and MC conducted experiments. JT and BAC conducted data analysis. JT, BAC, LJB, SDE, MZL, MC, and ESC contributed to figures and data presentation. JT, BAC, and ESC wrote the manuscript. All authors edited and provided feedback on the final draft of the manuscript.

## Conflict of interest

The authors have declared that no conflict of interest exists.

## Funding support

This work is the result of NIH funding, in whole or in part, and is subject to the NIH Public Access Policy. Through acceptance of this federal funding, the NIH has been given a right to make the work publicly available in PubMed Central.

- NIH (grants DA042111 and DA048931 to ESC, DA056221 to BAC).
- The Brain and Behavior Research Foundation (to ESC).
- The Whitehall Foundation (to ESC).
- The Edward Mallinckrodt Jr. Foundation (to ESC).

## Supplementary Material

Supplemental data

Unedited blot and gel images

Supporting data values

## Version 1 03/17/2026

In-Press Preview

## Version 2 05/15/2026

Electronic publication
