# Supplementary material for: Sex and ovarian hormone cycles alter effects of stimulant drugs on mouse dopaminergic signaling
Source: J Clin Invest. 2026 Mar 17;136(10):e178630. doi: 10.1172/JCI178630 (PMC13178643; doi:10.1172/JCI178630)
Supplement: Supplemental data [file jci-136-178630-s130.pdf]

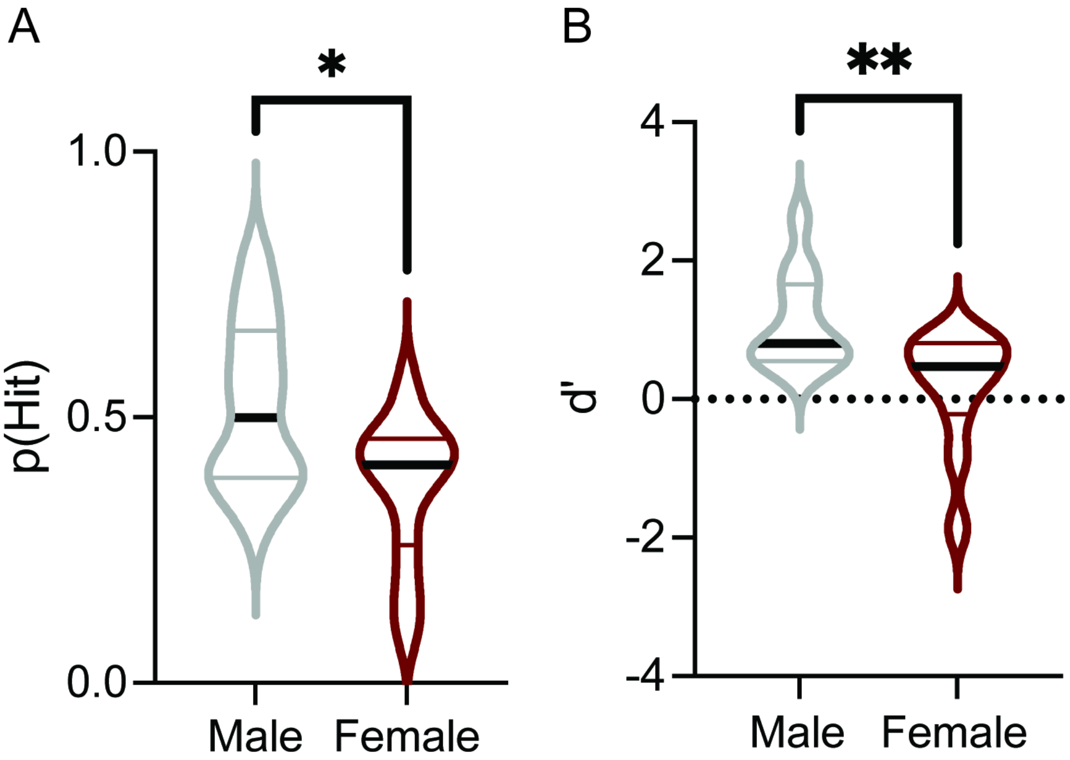


**Supplementary Figure 1. Sex differences in baseline behavior in modified psychomotor vigilant task.** Males demonstrated a higher **(A)** hit rate [Unpaired t-test: t (21) = 2.45, p = 0.023] and **(B)** d' [Unpaired t-test: t (21) = 2.89, p = 0.0088]. Data are presented as mean ± SEM. * p < 0.05, **p < 0.01.

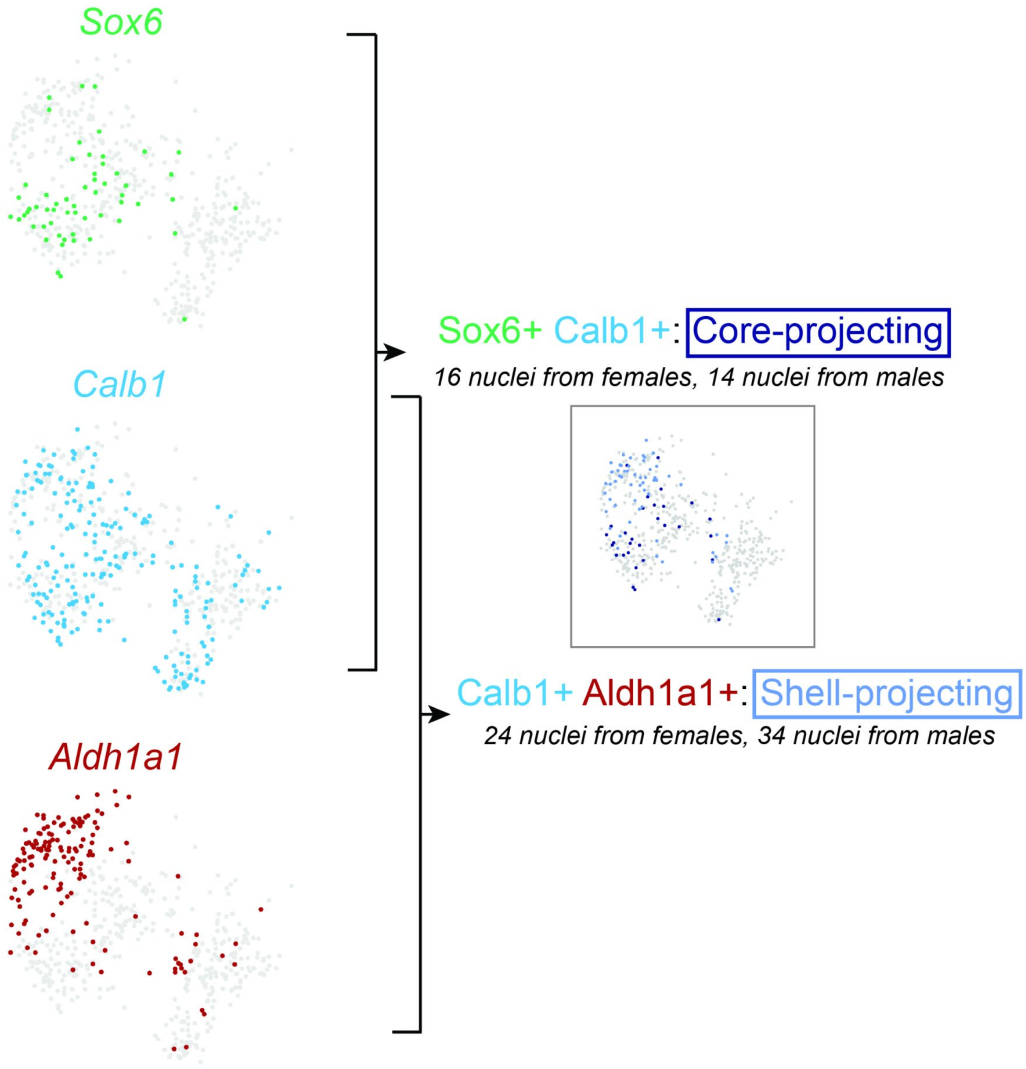


**Supplementary Figure 2. Characterization of core- vs shell-projecting dopamine neurons.** Marker gene–projection target mapping was used as described in Poulin et al. (2018) to define NAc core- and shell-projecting neurons. Core-projecting neurons were identified as nuclei co-expressing Calb1 and Sox6, while shell-projecting neurons were identified as nuclei co-expressing Calb1 and Aldh1a1.

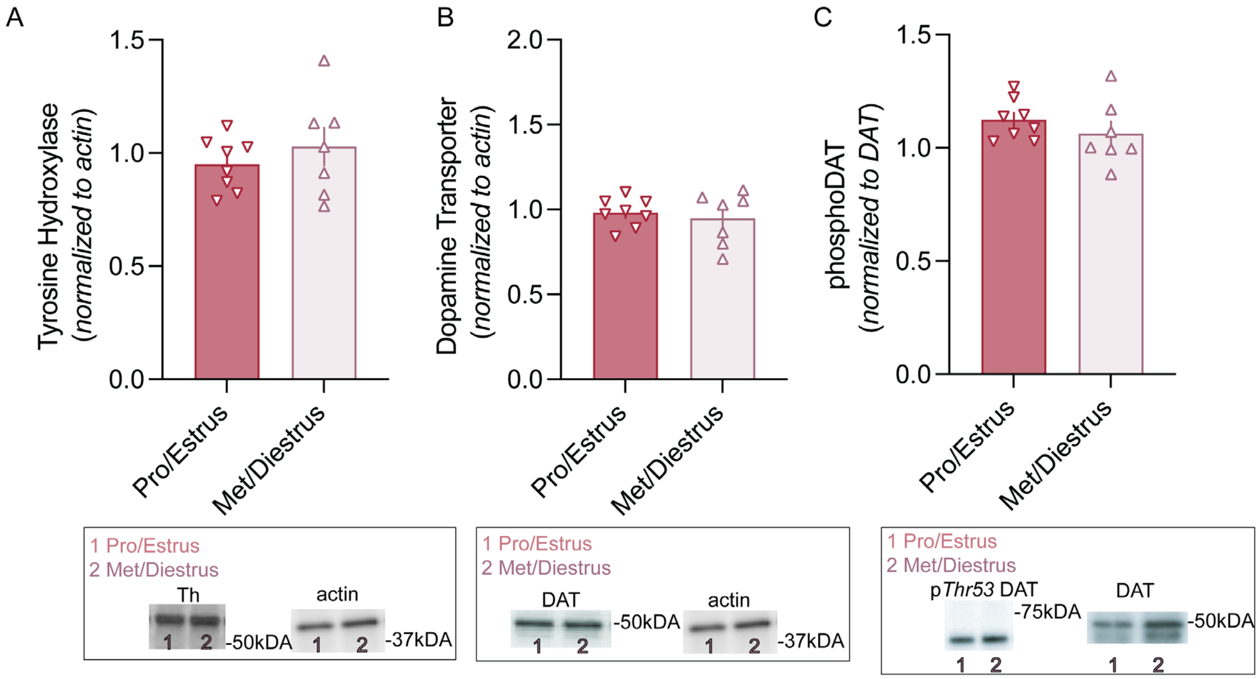


**Supplementary Figure 3. Protein expression in NAc terminals over the estrous cycle.** Protein expression in NAc core tissue punches was assessed via western blot analysis in pro/estrus and met/diestrus adult mice. There were no differences across cycle stage in the expression of **(A)** tyrosine hydroxylase [Unpaired t-test: t (13) = 0.87, p = 0.398], **(B)** dopamine transporter [Unpaired t-test: t (13) = 0.52, p = 0.61], or **(C)** p*Thr53* DAT [Unpaired t-test: t (13) = 1.013, p = 0.33]. Note: the same actin image is reused in panel A and B for comparison. Data are presented as mean ± SEM. * p < 0.05, **p < 0.01, ****p < 0.0001.

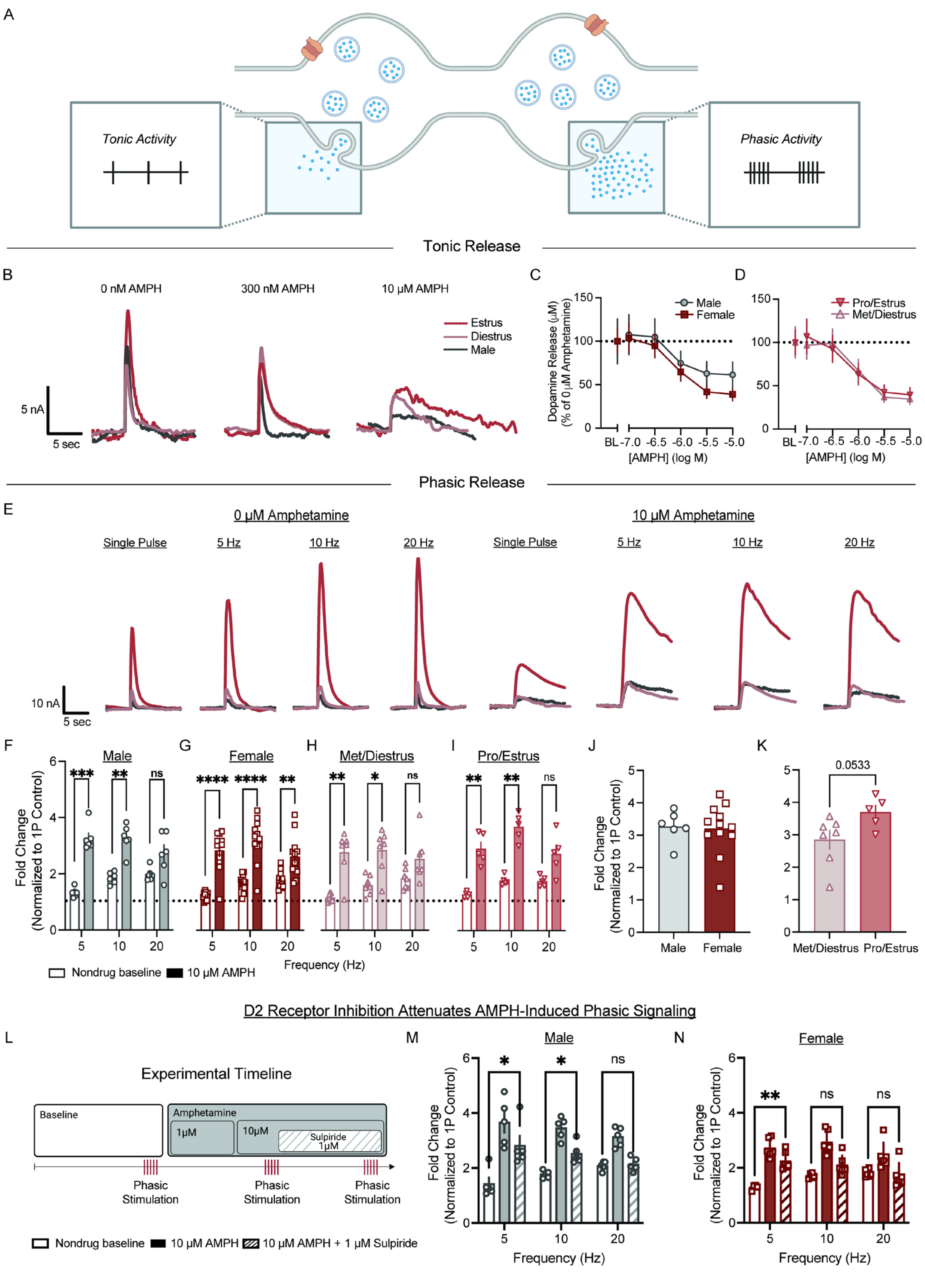

A
Tonic Activity
Phasic Activity
Tonic Release
B
0 nM AMPH
300 nM AMPH
10 µM AMPH
Estrus
Diestrus
Male
5 nA
5 sec
C
Male
Female
Dopamine Release (µM) (% of 0 µM Amphetamine)
[AMPH] (log M)
D
Pro/Estrus
Met/Diestrus
Phasic Release
E
0 µM Amphetamine
10 µM Amphetamine
Single Pulse
5 Hz
10 Hz
20 Hz
10 nA
5 sec
F
Male
G
Female
H
Met/Diestrus
I
Pro/Estrus
J
K
0.0533
Fold Change (Normalized to 1P Control)
Frequency (Hz)
Nondrug baseline
10 µM AMPH
D2 Receptor Inhibition Attenuates AMPH-Induced Phasic Signaling
L
Experimental Timeline
Baseline
Amphetamine
1µM
10µM
Sulpiride 1µM
Phasic Stimulation
M
Male
N
Female
Nondrug baseline
10 µM AMPH
10 µM AMPH + 1 µM Sulpiride

**Supplementary Figure 4. Amphetamine paradoxically decreases dopamine release in response to tonic stimulation parameters, while increasing phasic release.**

**(A)** Diagram depicting endogenous firing patterns in dopamine neurons. Tonic firing is characterized by slow, irregular firing (left), while phasic firing consists of short bursts of spikes that produce larger amounts of dopamine release (right). To mimic these effects, stimulation parameters were applied to slices to evoke release.

**(B-D) Tonic measurements.**

**(B)** Current versus time plots showing the effects of increasing concentrations of amphetamine on electrically evoked dopamine release in each group. Amphetamine decreased evoked release across a concentration–response curve, with no differences based on **(C)** sex [Mixed-effects analysis: main effect of drug, $F(1.576, 29.95) = 17.41$, $p < 0.0001$; no main effect of sex, $F(1, 20) = 0.178$, $p = 0.68$; no drug × sex interaction, $F(5, 95) = 0.39$, $p = 0.85$] or **(D)** estrous cycle stage [Mixed-effects analysis: main effect of drug, $F(1.952, 26.55) = 29.13$, $p < 0.0001$; no main effect of cycle stage, $F(1, 14) = 0.0012$, $p = 0.97$; no drug × cycle stage interaction, $F(5, 68) = 0.32$, $p = 0.90$].

**(E-K) Phasic measurements.**

**(E)** Current versus time plots showing the effects of 1 pulse and 5 pulses at stimulation frequencies of 5, 10, and 20 Hz at baseline (left) and following bath application of amphetamine (right) in each group.

**(F–I)** Group data showing amphetamine's effects on dopamine release across stimulation parameters, normalized to the single-pulse control within each group: **(F)** males [two-way repeated-measures ANOVA: main effect of amphetamine, $F(1, 10) = 51.28$, $p < 0.0001$; frequency × amphetamine interaction, $F(2, 20) = 9.33$, $p = 0.0014$]; (**G**) females [two-way repeated-measures ANOVA: main effect of amphetamine, $F(1, 22) = 49.75$, $p < 0.0001$; frequency × amphetamine interaction, $F(2, 44) = 8.68$, $p = 0.0007$]; **(H)** females in metestrus/diestrus [two-way repeated-measures ANOVA: main effect of stimulation, $F(1, 12) = 18.37$, $p = 0.0011$; stimulation × amphetamine interaction, $F(2, 24) = 5.62$, $p = 0.0100$]; **(I)** females in proestrus/estrus [two-way repeated-measures ANOVA: main effect of amphetamine, $F(1, 8) = 48.52$, $p = 0.0001$; stimulation × amphetamine interaction, $F(2, 16) = 5.32$, $p = 0.017$]. Amphetamine increased the ratio of phasic responses to tonic responses in all groups. **(J)** This effect did not differ between sexes [Unpaired t-test: $t(14) = 0.15$, $p = 0.88$]; however, **(K)** a trend was observed for greater fold change during pro/estrus compared with met/diestrus [Unpaired t-test: $t(10) = 2.19$, $p = 0.053$].

**(L-N)** Defining the mechanism contributing to AMPH-induced enhancement of phasic release. We tested the hypothesis that this enhancement of phasic/tonic dopamine release originated from AMPH-induced elevations in extracellular dopamine that activate $D_2$ autoreceptors, dampening tonic release but allowing high-frequency stimulation to overcome this inhibition. **(L)** AMPH was applied to slices in the presence of the D2 receptor antagonist sulpiride (1 μM). **(M–N)** Dopamine release was measured during tonic (1 pulse) and phasic (5–20 Hz) stimulations in males [two-way ANOVA: main effect of drug, $F(1, 8) = 52.94$, $p < 0.0001$, main effect of stimulation x drug interaction, $F(2, 16) = 6.39$, 0.0091] **(M)** and females [two way ANOVA: main effect of drug, $F(2, 15) = 21.98$, $p < 0.0001$, main effect of stimulation x drug interaction, $F(4, 30)$, 11.64, $p < 0.0001$ ] **(N)**. Sulpiride partially reduced—but did not abolish—AMPH-induced increases in dopamine release. In males, AMPH significantly enhanced release at 5 Hz and 10 Hz, while in females, significant enhancement was observed only at 5 Hz. Data are presented as mean ± SEM. *$p < 0.05$, **$p < 0.01$, ****$p < 0.0001$.

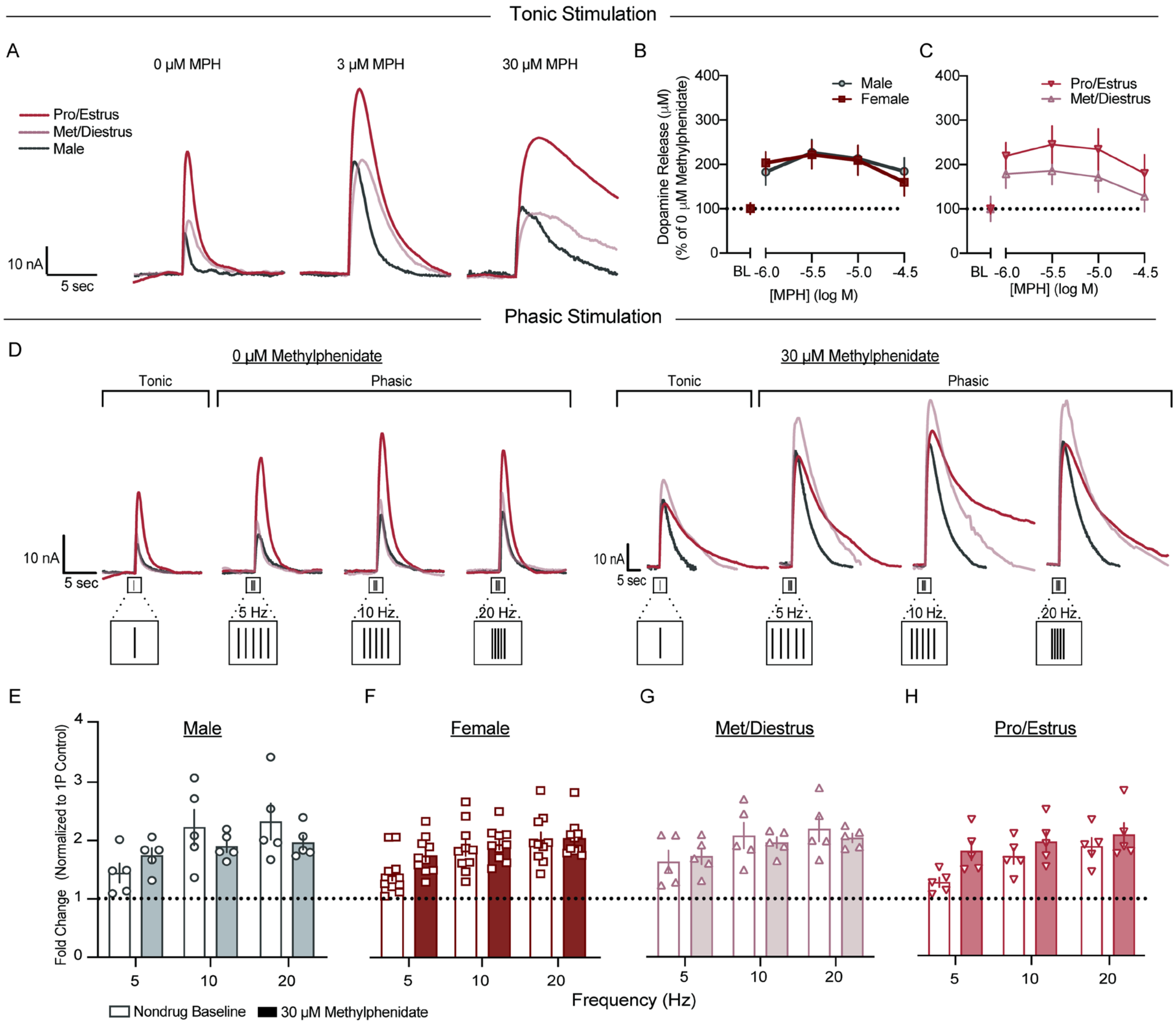


**Supplementary Figure 5. Methylphenidate effects on dopamine release are not sex- or cycle stage–dependent.**

**(A-C) Tonic measurements.**

**(A)** Current versus time plots showing the effects of increasing concentrations of methylphenidate on electrically evoked dopamine release in males (gray), females in proestrus/estrus (red), and females in metestrus/diestrus (pink). **(B–C)** Group data showing no differences in electrically evoked dopamine release based on **(B)** sex [Mixed-effects analysis: main effect of methylphenidate, $F(1.77, 27.46) = 17.14$, $p < 0.0001$; no main effect of sex, $F(1, 16) = 0.015$, $p = 0.90$; no methylphenidate × sex interaction, $F(4, 62) = 0.53$, $p = 0.71$] or **(C)** cycle stage [Mixed-effects analysis: main effect of methylphenidate, $F(1.316, 11.84) = 10.70$, $p = 0.0045$; no main effect of cycle stage, $F(1, 9) = 1.030$, $p = 0.34$; no dose × cycle stage interaction, $F(4, 36) = 0.77$, $p = 0.55$].

**(D-H) Phasic measurements.**

**(D)** Current versus time plots showing the effects of 1 pulse and 5 pulses at stimulation frequencies of 5, 10, and 20 Hz at baseline (left) and following methylphenidate application

(right) in each group. **(E–H)** Group data showing the effects of methylphenidate on electrically evoked dopamine release across stimulation parameters (5 Hz, 5 pulses; 10 Hz, 5 pulses; 20 Hz, 5 pulses), normalized to the single-pulse control in each group: **(E)** males [two-way repeated-measures ANOVA: no main effect of methylphenidate, $F(1, 8) = 0.23$, $p = 0.64$; stimulation × methylphenidate interaction, $F(2, 16) = 5.629$, $p = 0.0141$]; **(F)** females [two-way repeated-measures ANOVA: no main effect of methylphenidate, $F(1, 18) = 0.81$, $p = 0.38$; stimulation × methylphenidate interaction, $F(2, 36) = 5.933$, $p = 0.0059$]; **(G)** females in metestrus/diestrus [two-way repeated-measures ANOVA: no main effect of methylphenidate, $F(1, 8) = 0.078$, $p = 0.79$; no stimulation × methylphenidate interaction, $F(2, 16) = 2.22$, $p = 0.14$]; **(H)** females in proestrus/estrus [two-way repeated-measures ANOVA: no main effect of methylphenidate, $F(1, 8) = 2.797$, $p = 0.133$; no stimulation × methylphenidate interaction, $F(2, 16) = 3.16$, $p = 0.067$]. Data are presented as mean ± SEM.

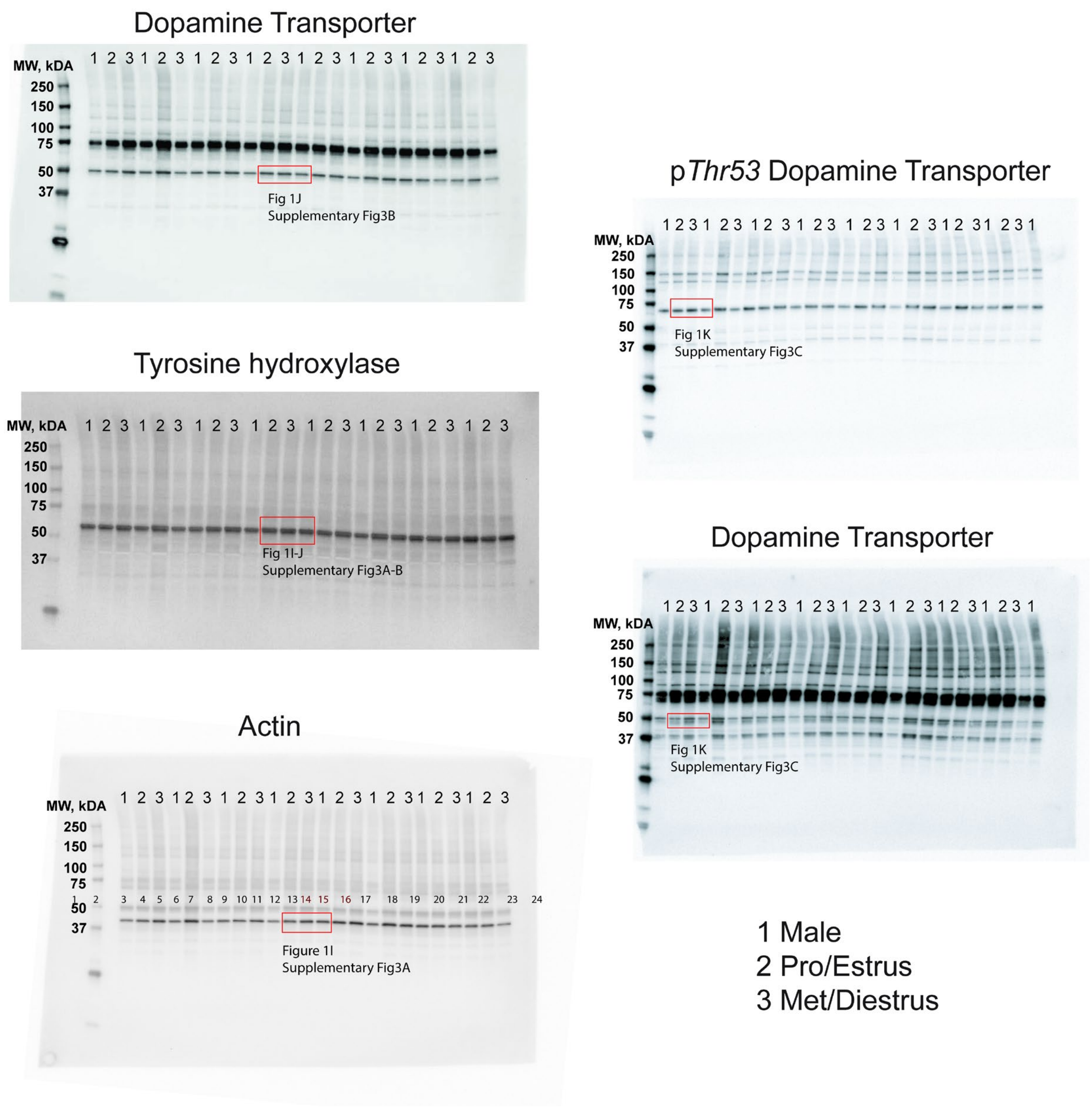


**Supplementary Figure 6. Full western blots.** Red boxes represent the representatives selected for main figures as noted.
