# Supplementary figures and images for: Sex and ovarian hormone cycles alter effects of stimulant drugs on mouse dopaminergic signaling
Source: J Clin Invest. 2026 Mar 17;136(10):e178630. doi: 10.1172/JCI178630 (PMC13178643; doi:10.1172/JCI178630)

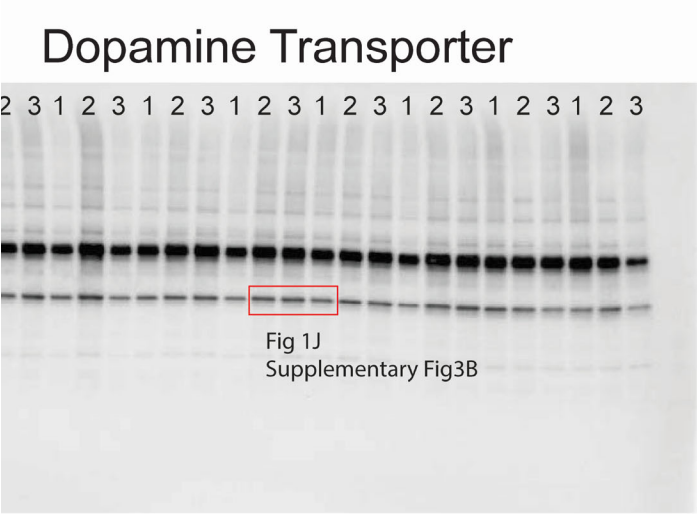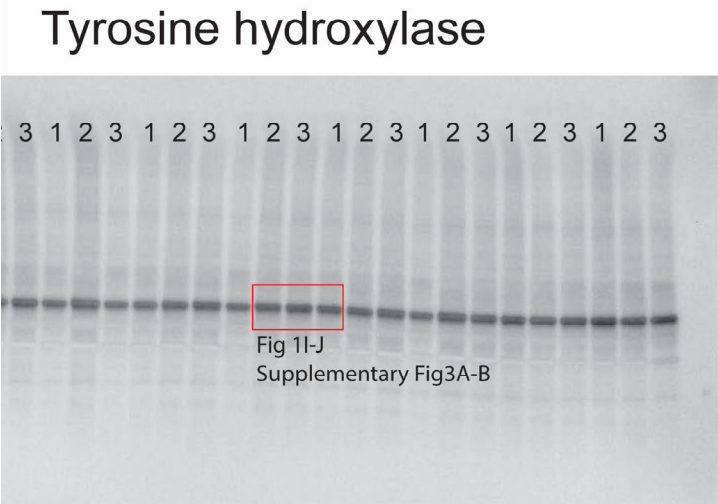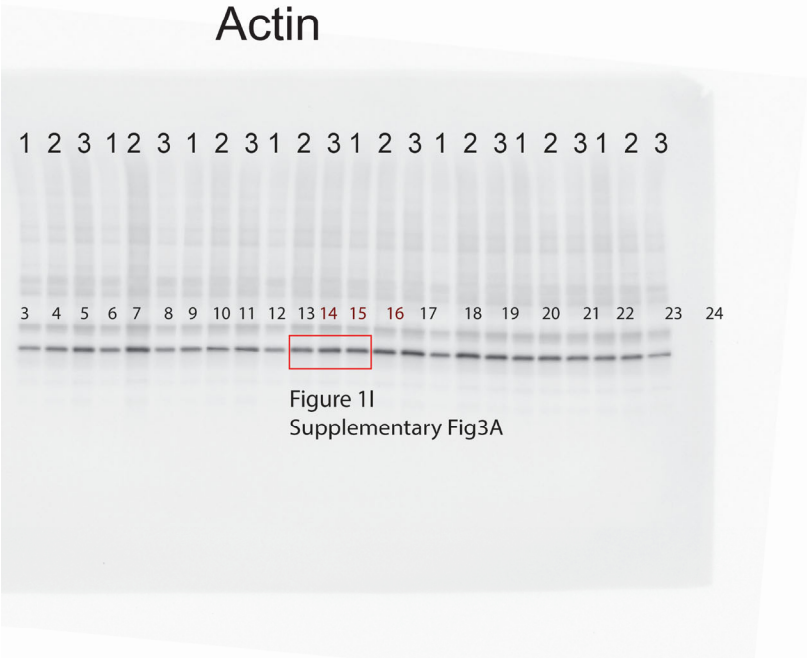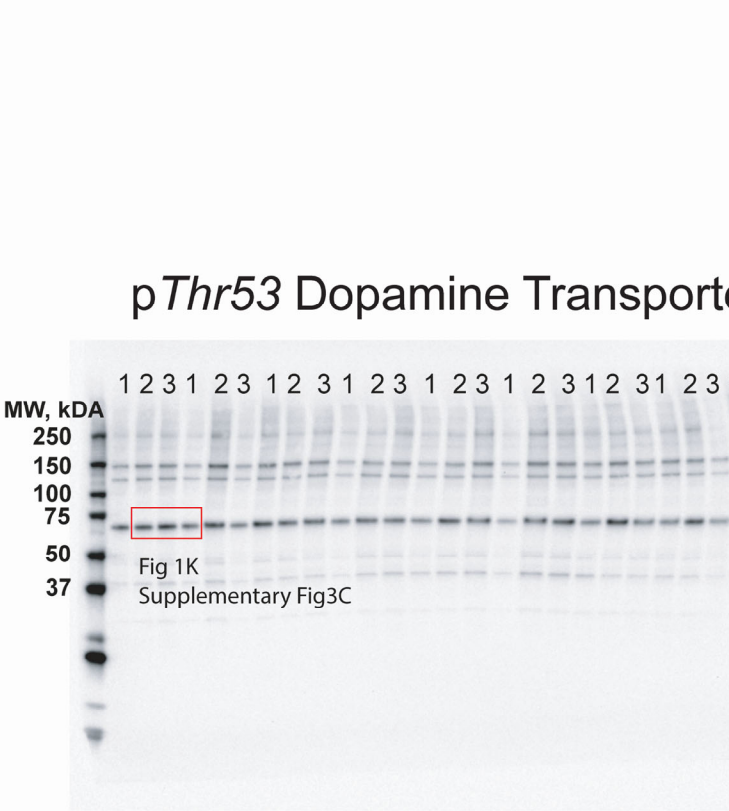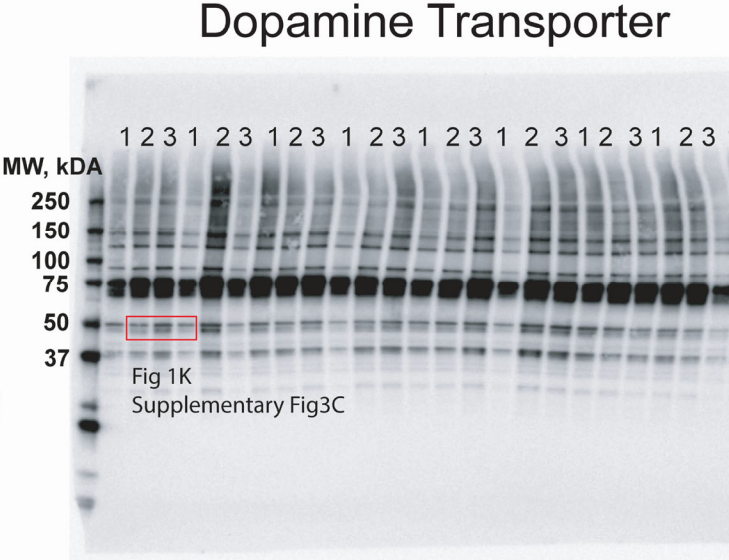

1 Male  
2 Pro/Estrus  
3 Met/Diestrus

Supplement: Unedited blot and gel images [file jci-136-178630-s131.pdf]
